# Bioactivity of Decavanadate Compounds: Can Their *In Vitro* and *In Vivo* Effects Be Assessed in a Simple Manner?

**DOI:** 10.1021/acs.inorgchem.5c03076

**Published:** 2025-10-16

**Authors:** João Costa Pessoa, Rim Zarroug, Nádia Ribeiro, Isabel Correia, Leonor Corte-Real, Brahim Ayed, Clara S. B. Gomes, Fernanda Marques, Albert Masip-Sánchez, Marina Hernández-Carrasco, Xavier López

**Affiliations:** 1 Centro de Química Estrutural, Institute of Molecular Sciences and Departamento de Engenharia Química, Instituto Superior Técnico, Universidade de Lisboa, Av. Rovisco Pais, Lisboa 1049-001, Portugal; 2 Laboratory of Physico-chemistry of Materials LR01ES19, Faculty of Sciences of Monastir, University of Monastir, Monastir 5000, Tunisia; 3 Department of Chemistry, Faculty of Sciences, University of Gabes, Gabes 6072, Tunisia; 4 LAQV-REQUIMTE, Department of Chemistry, NOVA School of Science and Technology, NOVA University Lisbon, Campus de Caparica, Caparica 2829-516, Portugal; 5 UCIBIO, Department of Chemistry, NOVA School of Science and Technology, NOVA University Lisbon, Campus de Caparica, Caparica 2829-516, Portugal; 6 Associate Laboratory i4HB, NOVA School of Science and Technology, NOVA University Lisbon, Campus de Caparica, Caparica 2829-516, Portugal; 7 Centro de Ciências e Tecnologias Nucleares and Departamento de Engenharia e Ciências Nucleares, Instituto Superior Técnico, Universidade de Lisboa, Estrada Nacional 10, Bobadela LRS 2695-066 Portugal; 8 Departament de Química Física i Inorgànica, 16777 Universitat Rovira i Virgili, Marcel·lí Domingo 1, Tarragona 43007, Spain

## Abstract

Two decavanadate anions with 4-dimethylaminopyridinium and one with 1-methylimidazolium cations are isolated and characterized by single-crystal X-ray diffraction. The Hirshfeld surfaces and associated 2D-fingerprint plots confirm the propensity of V_10_ anions to undergo hydrogen-bonding interactions. DFT calculations demonstrate the tendency for protonation of decavanadates and, for the first time, the possibility of one or two-electron reduction maintaining the V_10_-cluster structure. Moreover, the tendency of V_10_ species to gain electrons increases with ion pairing, this effect depending on the counterion involved. The computed proton-coupled electron transfer energies indicate the prevalence of [HV^V^_9_V^IV^O_28_]^6–^, [H_2_V^V^_8_V^IV^_2_O_28_]^6–^, or [H_2_V^V^_9_V^IV^O_28_]^5–^ species over V_10_. Decavanadates at total vanadium concentrations ([V]_total_) in the range 720–800 μM undergo extensive hydrolysis within a few hours when added to RPMI cell incubation media. With the cell media in contact with A2780 ovarian cancer cells, V_10_ hydrolysis occurs much faster. Notably, the cytotoxicity and the vanadium uptake observed on A2780 cells for equal [V]_total_ values are similar to solutions containing or not V_10_ anions, but preincubation with the V^V^ solutions affect the biological activities, decreasing cell viability. These conclusions support the complexity of factors to be analyzed when discussing any biological effect observed, namely, protonation, redox processes, hydrolysis, ageing, counterions, and composition of the biological medium.

## Graphical abstract

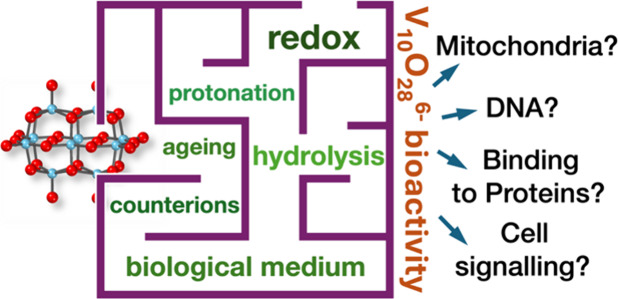

## 1 Introduction

Polyoxidometalates (POMs) are a class of inorganic, mainly anionic clusters that contain several transition metal centers (such as tungsten, molybdenum, and vanadium) possessing a wide variety of sizes and geometric features. In the so-called mixed-valence compounds, not all the metal centers feature the same oxidation state. POMs have been extensively studied due to their potential applications in various fields such as environmental science, catalysis,^1–7^ materials science,^7–9^ and therapeutics.^10–27^ The nature of the metal centers, the molecular size, and the structure are among the important characteristics that determine their properties, hence the type of applications encompassed for each POM. Among the wide variety of POMs, the family of polyoxidovanadates (POV) includes, among others, di- (V_2_, H_n_V_2_O_7_^–4+*n*^), tetra- (V_4_, HV_4_O_13_^5–^, V_4_O_12_^4–^), penta- (V_5_, V_5_O_15_^5–^), and decanuclear (V_10_, H_n_V_10_O_28_^–6+*n*^) species.^16,20,28–34^

The decavanadate species (V_10_, H_n_V_10_O_28_^–6+*n*^) are important compounds that have been studied extensively in the past decades because of their putative catalytic properties^3,5,18,35,36^ and versatile bioactivity.^10–23,10–23,26–32,10–23,26–32,37–40^ In aqueous solution and at mM concentrations, decavanadates are stable in the pH range ca. 3.5–5.5 but, outside this interval, or at μM concentrations, they decompose forming smaller POVs and monovanadate (V_1_). The particular structures that may be present in solution or isolated in the solid state depend on the solvent, pH value, ionic strength, temperature, and nature of the counterions present.^33,34,38,40,33,34,41–48^

One approach that is often used is to characterize the systems in conditions where decavanadates are thermodynamically and/or kinetically stable, e.g., 1–50 mM of total vanadium­(V) concentrations ([V^V^]_total_), assumes that the same type of species predominate at μM levels. However, speciation of V-containing species at low [V^V^]_total_ or in biological media may be quite different. In this work, we characterize decavanadate systems both at relatively high vanadium concentrations ([V^V^]_total_ = 1–20 mM) but also at much lower values and at pH ∼ 7. The studies globally indicate that at pH ∼ 7 and with most vanadium concentrations employed, several V^V^-species coexist, and it is not trivial to isolate the putative action of each of them and/or consider that the biological effect observed can be attributed in a simple and/or direct manner to one of the oxidovanadate­(V) species present. Certainly, these facts have been known for many years but have not been properly considered in many of the studies reported.

One of the most frequently mentioned biological effects of vanadium compounds on cells is the excessive production of reactive oxygen species (ROS) that cannot be counteracted by the available antioxidant defenses,^21,34^ and the possibility of easy interchange between the V^IV^ and V^V^ states in the vanadium-based system under consideration is often mentioned as being relevant for the biological activity.^21,34,49,50^ Namely, if a particular system is able to support the efficient Fenton-like conversion of V^IV^ and V^V^ species, the formation of HO^•^ via H_2_O_2_ may become excessive and destroy the structure or integrity of cells, leading to apoptosis. For example, the conversion between V^IV^ and V^V^ may lead to consumption of glutathione (or other biological reducing agents), and besides other possible effects, if fatty acids within the membrane undergo peroxidation, this may disrupt the membrane structure leading to increased permeability or leaky membranes. Therefore, one approach for treatment of cancer has been the design of V-containing nanoparticles, which ensure that the energy band gap between V^IV^ and V^V^ species is adequate for efficient production of HO^•^ inside cancer cells.^34,51–54^

Mixed-valence POVs have also been studied, and such V^IV^/V^V^ clusters are interesting compounds in this context. In fact, several mixed-valence V_6_, V_10_, V_12_, V_15_, V_18_, V_19_, and V_22_ POVs have been characterized by single-crystal X-ray diffraction (SC-XRD).^48,55–57^ For example, in ((C_2_H_5_)_4_N)_4_[V^IV^_2_V^V^_8_O_26_]·H_2_O, the V_10_ anion has a crown-like shape of eight V^V^O_4_ tetrahedra to which two V^IV^O_5_ square pyramids are appended, but its structure is quite distinct from the typical decavanadates,^55^ where the V_10_ anions exhibit a cage-like structure, with the ten V^V^ centers arranged in space in three layers. In some cases, the preparation of mixed-valence V^IV^/V^V^ polyoxidovanadates involved the replacement of some of the oxido ligands in the parent structure by alkoxido donors,^58–61^ some of these examples being of decavanadate clusters; to our knowledge, these are the only examples of V_10_ mixed-valence V^IV^/V^V^ clusters, characterized by SC-XRD, where the typical decavanadate structure was maintained.^60–62^

The biological properties of the V_10_ anions might be associated with their redox properties. These have been scarcely studied, and in some cases, redox processes were considered to result either in decomposition of the V_10_ clusters^34^ or in changes of their structure. In one case, the cyclic voltammograms of two V_10_ salts at 1–6 mM concentrations were measured in aqueous solution with KNO_3_ as supporting electrolyte. In both cases the voltammograms were consistent with a quasi-reversible redox behavior, with *E*_1/2_ of 180 and −300 mV (relative to saturated Ag/AgCl electrode), which were attributed to V^V^/V^IV^ redox couple processes centered on the V_10_ core; the different *E*_1/2_ values obtained were justified by the presence of distinct counterions.^63^ As rather similar electrochemical behavior was observed for several V_4_ salts,^64^ whether the integrity of the clusters is maintained or not cannot be confirmed. Therefore, a better understanding of the redox behavior of the V_10_ anions is required.

Upon contact with a vanadium compound, cells may uptake V-containing species; however, the total vanadium concentration inside cells is normally very low and a significant part of it is normally considered to be present as V^IV^O-species and probably part bound to proteins.^21,34,65–81^ Therefore, the intracellular concentration of V^V^-species is typically very low, the main species formed probably being H_2_V^V^O_4_^–^ and HV^V^O_4_^2–^ ^21,42,81^ ; thus, POVs such as V_10_ anions are not expected to form. Nevertheless, it has been stated that particular conditions may favor the formation of POV anions, namely, decavanadates, *in vitro* or *in vivo*.^8,16,20,23,34,63,65,68^ In fact, it is conceptually possible that V_10_ anions may form in cellular compartments containing V^V^-species, and cytosol, mitochondria, and acidic organelles have been mentioned as probable examples.^16^ Notably, it has been shown that upon interaction with proteins, solutions containing vanadium salts or compounds may form adducts of different nuclearity.^64–86^ Several biological activities and/or reports of interaction with proteins have been associated with polyoxidovanadate­(V) species, namely, with decavanadates.^17–23,31,39,63,17–23,82–86^ For example, V_2_ was reported to influence the activity of isomerases, hydrogenases, aldolases, and phosphatases,^26,81^ and V_4_ was reported as an inhibitor of aldolases and dehydrogenases^81,86^ as well as photocleavage of myosin.^87,88^ Some decavanadate compounds have been reported to show insulin enhancing activity,^27,40,85,89^ to induce depolarization of mitochondria,^90^ as being the active species in photocleavage of myosin at phosphate binding sites,^91^ as activators of nucleotidases,^92^ as inhibitors of kinases,^93^ and as inhibitors of myosin ATPase activity stimulated by actin and muscle phosphorylases,^39^ as well as initiators of cell signaling.^74,94^ However, besides difficulties in transport across cell membranes, which may be anticipated to arise from their large negative charge and size, in many cases, speciation of decavanadates in the media where experiments were carried out was not properly evaluated or even considered. As mentioned above, the V^V^ concentration and pH conditions determine the type of vanadate­(V) species present and thus affect the vanadium biological activity. The presence of V_10_ anions may be favored in particular membrane or cell sites, where vanadium may possibly accumulate and/or be protected from hydrolysis through hydrogen-bonding interactions with surrounding molecules, with some proteins being candidates for such interactions. Notwithstanding, it is feasible that V_1_, V_2_, V_4_, and/or V_5_ anions may also be involved in favorable interactions with the same or other biological sites or molecules. To our best knowledge, it was also never discussed if, e.g., redox reactions of POVs bound to cell membranes (or relevant biomolecules) may induce cell signaling or/and relevant bioactivity.

Organic cations often improve the solubility of the POVs. Additionally, several of them may bind either covalently or noncovalently with surfactants, and this may possibly improve their bioavailability for different biological functions. Additionally, those able to establish hydrogen bonding allow the possibility of obtaining new interesting decavanadate clusters with improved versatility in the binding to relevant biomolecules. Among our objectives was to evaluate if the two organic cations hereby used (4-dimethylaminopyridinium and 1-methylimidazolium) interact with V_10_ in aqueous solution, affect the redox characteristics of V_10_ anions, and/or if they change the cytotoxicity of decavanadate.

Anticipating possible electrostatic, dipolar, or hydrogen-bonding interactions, several polyoxidovanadate­(V) compounds containing organic cations have been synthesized and have shown interesting pharmacological activities, suggesting their potential therapeutic use.^27,30,32,38,40,46,47,63,46,47,95–104^ However, how the organic cations participate in the biological activity is normally not made clear. Moreover, some of them possibly are bioactive, namely, they display toxic effects, these depending on several factors such as the mode of administration, type of tissue or cell fraction, concentration used, and exposure time. In this context, a phosphotetradecavanadate (PV_14_) compound with benzylammonium cations as counterions was recently reported.^30^ While in the solid state, these organic cations are bound to the POV, in solution, even at mM concentrations, they are not significantly associated with the PV_14_ anions, raising doubts if they may be relevant for the bioactivity of the PV_14_ moiety. Regarding V_10_ salts with organic cations, it has been often stated that these counterions may in some way potentiate favorable biological effects of V_10_ anions. It is a fact that in the solid state, several of these cations are involved in strong interactions with the decavanadate clusters, but it is not known if such interactions are preserved in solution, particularly at low concentrations. Moreover, to our knowledge, it was never clearly shown if organic cations promote or not interactions of POV anions with cell membranes or with biologically relevant molecules.

Hereby, we report the preparation and full characterization in the solid state, including single-crystal X-ray diffraction (SC-XRD) of three new decavanadate polyanion salts containing organic cations. Two of them were with 4-dimethylaminopyridinium and one with 1-methylimidazolium. The noncovalent interactions are evaluated for the new decavanadate compounds by Hirshfeld surface analysis (HS) and we also evaluate if in solutions with average and low V^V^ concentrations, the organic cations might be significantly associated with the V_10_ anions or not. The experimental measurements are supplemented with density functional theory (DFT) and molecular dynamics (MD) calculations, which provide valuable information at the molecular level about the behavior of the vanadate species in aqueous solution in the presence of the two cations herein reported. In addition, the dependence of protonation and electron transfer processes on POV-cation pairing is analyzed.

Several cytotoxicity experiments were carried out with A2780 ovarian cancer cells to compare the activity of solutions with equal total vanadium concentrations, containing or not containing decavanadates, as well as solutions containing V_10_ anions and 4-dimethylaminopyridine (4-Me2AmPy, this molecule is sometimes abbreviated as DMAP) or 1-methylimidazole (1-MeIm), or only containing the organic cations. ^51^V NMR experiments were also recorded with solutions of decavanadates in the cell incubation media, with and without added fetal bovine serum, as well as with the separated supernatant of the cell medium after its contact with A2780 cells.

Globally, our results highlight the complexity of the factors to be analyzed when trying to understand any particular biological effect observed in solutions containing decavanadates.

## 2 Experimental Section

### 2.1 Materials and Measurements

Sodium metavanadate (NaV^V^O_3_, Sigma) and acetic acid were used as provided. Millipore water was used in all experiments with biological macromolecules. All other materials were either p.a. or of equivalent purity.

The NMR spectra were recorded at ambient temperature on a Bruker Avance II + 500 (Ultra Shield TM Magnet) spectrometer operating at 500.13 MHz. The ^51^V NMR chemical shifts (δ_V_) are reported in ppm using neat V^V^OCl_3_ as reference. The infrared spectra were recorded on an Alpha RT-DLaTGS HR 0.8 FTIR spectrometer, and the UV–visible (UV–vis) absorption spectra were recorded on a PerkinElmer Lambda 35 UV–vis spectrophotometer with either 2.0 or 10.0 mm optical path cuvettes.

Unless otherwise specified, the concentrations of vanadium in solution always correspond to the total vanadium­(V) concentrations, [V]_total_, either if this concerns samples containing or not containing decavanadates; these solutions are specified as V_1_ or V_10_ (see below). If the vanadium is totally in the form of decavanadates, which will be globally designated by V_10_, then in these solutions, [V]_total_ = 10×[V_10_]. All solutions used for spectroscopic and biological experiments where decavanadates and organic cations are simultaneously present were prepared using solutions of V_1_ or V_10_, and the organic compound were added separately.

### 2.2 Chemical Preparation and Crystallization of Compounds **1**–**3**

#### Synthesis of (C_7_H_11_N_2_)_4_[H_2_V_10_O_28_] (**1**) and (**2**)

Compounds **1** and **2** were synthesized by dissolving NH_4_V^V^O_3_ (6 mmol) in hot water (30 mL, *t* ∼ 80 °C) in a round-bottom flask. 4-Dimethylaminopyridine (0.5 mmol) was added, and the pH was adjusted to 3.0 by dropwise addition of acetic acid (6 M). The mixture was stirred for 1 h and left to cool down to room temperature (ca. 27 °C). After slow evaporation at room temperature for several days, two types of small crystals, orange/bronze and yellow/bronze, were obtained and separated. Both are formulated as (C_7_H_11_N_2_)_4_[H_2_V_10_O_28_], but while **1** (around 80% of the total number of crystals) crystallized in the triclinic system and space group P-1, **2** crystallized in the monoclinic system and space group *P* 2_1_/*n.*

#### Synthesis of (C_4_H_7_N_2_)_6_[V_10_O_28_]·8H_2_O (**3**)

Compound **3** was synthesized by dissolving NH_4_V^V^O_3_ (1 mmol) in hot water (25 mL, *t* ∼ 80 °C) in a round-bottom flask. 1-Methylimidazole (0.1 mL) was added, and the pH was adjusted to ∼4 by dropwise addition of acetic acid (6 M). The mixture was stirred for 1 h and left to cool down to room temperature (ca. 27 °C). After evaporation at room temperature for 2 weeks, small orange crystals were obtained and separated.

Compounds **1**–**3** are moderately soluble in water. Elemental analysis. Compounds **1** and **2**: C_28_H_46_N_8_O_28_V_10_ (Exp/Ther) C: 23.02/23.16; H: 3.28/3.19; N: 7.61/7.72. Compound **3**: (Exp/Ther): C_24_H_58_N_12_O_36_V_10_ C: 17.88/18.01; H: 3.79/3.65; N: 10.36/10.50.

Safety concerns: 4-Dimethylaminopyridine. 4-Me2AmPy (toxic by ingestion, inhalation, and skin absorption; irritant) was handled exclusively in a fume hood while wearing nitrile gloves, a lab coat, and safety goggles. Waste was collected in designated hazardous containers. 1-Methylimidazole. 1-MeIm corrosive; harmful by inhalation, ingestion, or skin contact) was weighed and transferred in a fume hood with appropriate PPE (nitrile gloves, lab coat, and safety goggles). Waste solutions were segregated and disposed of according to hazardous waste protocols.

### 2.3 Preparation of Solutions for UV–vis and ^51^V NMR Spectroscopic Measurements

Millipore water and Hepes buffer ((4-(2-hydroxyethyl)-1-piperazineethanesulfonic acid, 5 mM, pH = 7.1 ± 0.1) were used in most experiments where a buffer of pH ∼ 7 was required. The concentrations of V^V^-containing solutions are expressed in terms of the total vanadium content. Stock solutions of decavanadate ([V]_total_ ≈ 50 mM, thus [V_10_] ≈ 5.0 mM; for each solution prepared the V^V^ concentration is accurately known) were prepared in 100.00 mL volumetric flasks by dissolving NaV^V^O_3_ (ca. 0.62 g, accurately weighted) in water. In each preparation, under stirring, water was added till ∼90% of total volume, and the pH was then adjusted to 4.0 using a 1.0 M HCl solution. Next, the total volume was set to 0.5 mL with water. These solutions will be designated as ‘V_10_ solution’, and the ^51^V NMR spectrum of one of these V_10_ stock solutions at pH ≈ 4.0 is depicted in Figure S1. Increasing the pH of this solution to ∼5.3 and recording the ^51^V NMR spectrum after ca. 10 min, the bands due to decavanadate species are the predominant ones, but peaks due to V_1_, V_2_, and V_4_ become clearly visible (Figure S2).

Stock solutions used for experiments in the absence of decavanadates were prepared by dissolving accurately weighted amounts of NaV^V^O_3_ at relatively high pH, for example, in NaOH ∼0.001 or 0.01 M. These solutions were next diluted 1:10 or 1:100 with water, the pH becoming ca. 10.0. Figure S3 depicts the ^51^V NMR spectrum of one of such stock solutions at pH = 10.0; it is clear that no decavanadate anions are detected. Solutions prepared in this way will be designated as ‘V_1_ solution’; they do not contain decavanadates but may contain small amounts of other V^V^-species. As during their preparation at no occasion was the pH below 7 in any portion of the solutions, V_10_ anions do not form. Similar caution was taken when using these solutions in the experiments.

Several spectroscopic measurements were carried out by adding the ‘V_10_ solution’ to a solution of Hepes buffer at pH ∼ 7.0 at several time points up to ∼48 h upon mixing the two solutions. The objective of these experiments was to evaluate the evolution with time of the relative concentrations of the several V^V^-species present.

Several ^51^V NMR spectral measurements were carried out adding the ‘V_10_ solution’ to RPMI-1640 (Gibco, Thermo Fisher Scientific) incubation media not containing or containing 2 or 10% fetal bovine serum (FBS). The [V]_total_ was 720 and/or 800 μM.

For each of these solutions containing 10% (v/v) of D_2_O, ^51^V NMR spectra were measured at ca. 1, 3, and ∼18 h after adding the ‘V_10_ solution’ to the RPMI-1640 media (containing or not FBS). Note that the RPMI-1640 media contains 23.8 mM sodium bicarbonate as a buffer (it does not contain Hepes) and ca. 11.1 mM d-glucose. Also in these experiments, their objective was to evaluate the evolution with time of the relative concentrations of the several V^V^-species present.

To RPMI-1640 incubation media containing 10% FBS in contact with ovarian A2780 cancer cells, ‘V_10_ solutions’ with [V]_total_ = 720 μM or 800 μM were added. After either 1 or 3 h, the cell media were separated from the cells and ^51^V NMR spectral measurements were carried out ∼1, ∼3, and ∼18 h after separation of the medium from the cells and addition of 10% (*v*/*v*) D_2_O. The objective of these experiments was also to evaluate the evolution with time of the relative concentrations of the several V^V^-species present.

### 2.4 Single-Crystal X-ray Diffraction

The most relevant crystallographic data for each compound and experimental details are presented in Table 1. Crystals suitable for single-crystal X-ray analysis of compounds **1**–**3** were selected, covered with Fomblin (poly­(fluoroether oil), and mounted on a nylon loop. The data were collected at 110(2) K on a Bruker D8 Venture diffractometer equipped with a Photon 100 CMOS detector and an Oxford Cryosystem Cooler, using graphite monochromated Mo–Kα radiation (λ=0.71073 Å). The data was processed using the APEX3 suite software package, which includes integration and scaling (SAINT), absorption corrections (SADABS),^105^ and space group determination (XPREP). Structure solution and refinement were done using direct methods with the programs SHELXT 2018/3 and SHELXL (version 2018/3)^106,107^ inbuilt in APEX, and WinGX-Version 2021.3 ^108^ software packages. Both crystals of **2** and **3** showed poorer quality and diffracting power, giving rise to low quality data. Multiple attempts were made to grow better diffracting crystals but revealed to be unsuccessful. Nevertheless, all characterization results are consistent with the remaining chemical characterization analysis and the model reported herein. The crystals of **1** and **2** showed the presence of disordered solvent molecules at room temperature, and the PLATON/SQUEEZE^109^ routine being applied as a good disorder model was impossible to attain. All non-hydrogen atoms were refined anisotropically. Except for NH and OH, the remaining hydrogen atoms were inserted in idealized positions and allowed to refine riding on the parent carbon atom. The molecular diagrams were drawn with ORTEP-3 (version 2020.1),^108^ included in the software package. The data was deposited in the CCDC under deposit numbers 2452234 for **1**, 2452235 for **2**, and 2452236 for **3.**

**Table 1:** Crystal Data and Structure Refinement for Compounds **1**, **2**, and **3**

	1	2	3
formula	C_28_H_46_N_8_O_28_V_10_	C_28_H_46_N_8_O_28_V_10_	C_24_H_58_N_12_O_36_V_10_
*M*	1452.13	1452.11	1600.22
λ (Å)	0.71073	0.71073	0.71073
*T* (K)	110(2)	110(2)	110(2)
crystal system	triclinic	monoclinic	monoclinic
space group	*P*-1	*P*2_1_/*n*	*P*2_1_/*n*
crystal description	prism	prism	prism
crystal color	bronze	yellow	yellow
*a* (Å)	9.8519(11)	15.623(3)	11.0603(15)
*b* (Å)	11.0820(11)	13.293(2)	16.454(2)
*c* (Å)	12.9098(14)	22.968(4)	14.7861(17)
α (deg)	68.433(3)	90	90
β (deg)	73.930(3)	95.720(6)	94.853(4)
γ (deg)	87.064(3)	90	90
*V* (Å^3^)	1257.6(2)	4746.1(14)	2681.2(6)
*Z*	1	4	2
ρ_calc_ (g cm^–3^)	1.917	2.032	1.982
μ (mm^–1^)	1.862	1.976	1.769
θ_max_ (deg)	35.296	27.962	33.936
total data	90291	74219	126145
unique data	11154	11371	10835
*R*_int_	0.1386	0.2063	0.2023
*R* [*I* > 3σ(*I*)]	0.0666	0.0612	0.0572
*wR*_*2*_	0.1455	0.1053	0.1122
goodness of fit	1.051	0.989	1.024
ρ_min_	–0.915	–0.719	–1.152
ρ_max_	0.192	0.155	0.182

### 2.5 Computational Details

#### Density Functional Theory (DFT) Calculations

All structures, including decavanadate (V_10_) species with varying degrees of protonation and reduction, as well as the V_1_, V_2_, and V_4_ subunits, were fully optimized using Gaussian 16 (Revision A.03)^110^ at the B3LYP-D3BJ^111–114^ level of theory. For vanadium atoms, we employed the LANL2DZ basis set^115^ augmented with Frenking’s f-type polarization functions,^116^ while main group elements were described using the 6–31G­(d,p) basis set.^117,118^ To account for solvation effects, particularly important when comparing systems with different molecular charges, we used the IEF-PCM implicit solvent model with the dielectric constant ε = 78.39 for water.^119^ A standard-state correction of +1.99 kcal mol^–1^ was applied to the computed free energies to account for the difference between the gas-phase reference state (1 atm) used in Gaussian thermochemistry and the solution-phase standard state (1 M) at 37 °C. Protonation free energies were computed using a value of −264.0 kcal mol^–1^, i.e., the experimental standard Gibbs free energy of a proton in aqueous solution.^120^ To assess the influence of counter cations on the electronic structure of V_10_, we selected five representative snapshots from molecular dynamics simulations that included the positions of the counter cations. DFT calculations were then performed for each snapshot, and the results were averaged to obtain a representative electronic profile, as done previously in other publications.^121,122^ Each snapshot was fully optimized and verified as a minimum on the potential energy surface with the H­(cation)–O­(POM) distance constrained throughout the process. A complete data set of the computational results is available from the ioChem-BD repository at 10.19061/iochem-bd-2-82.^123^

#### Molecular Dynamics (MD) Simulations

To investigate the distribution of cations around the polyoxidometalates in aqueous solution, we performed atomistic MD simulations with explicit solvent using GROMACS 2019.3.^124,125^ A modified version of the AMBER99 force field, previously validated for studying POM aggregation in diverse environments,^126^ was employed. The force field accounts for the system’s potential energy as the sum of bonded interactions (bond stretching, angle bending, and dihedral torsions) and nonbonded interactions. The latter are described by pairwise additive 1–6–12 Lennard-Jones and electrostatic potentials, considering interactions between atoms separated by more than three bonds. Parameters for the POV species were derived following the protocol of López et al.,^127^ as implemented in the topoMOx code.^128^ Water molecules were modeled using the TIP3P representation.^129^ Partial atomic charges were computed at the same level of theory as for the DFT calculations, employing the ChelpG method based on the electrostatic potential.

MD trajectories were generated under fully periodic boundary conditions within a cubic simulation box of 8.06 nm per side (∼512 nm^3^), containing five POV anions, the corresponding number of counterions to ensure electroneutrality, and a sufficient amount of explicit water molecules to achieve a final [V_10_] = 16 mM. Systems containing the V_1_, V_2_, and V_4_ species included 50, 25, and 12 POV units, respectively, corresponding to 50 vanadium centers in each case. A 10 Å cutoff was used for both van der Waals and short-range Coulombic interactions; the latter was further treated using the particle–particle mesh Ewald (PME) method to incorporate long-range electrostatics. Bond constraints involving hydrogen atoms were applied by using the LINCS algorithm.

Production simulations were conducted in the canonical (*NVT*) ensemble for 40 ns, with trajectory data collected for the last 20 ns every 1 ps. All simulations were performed at 310 K, maintaining the temperature via velocity-rescaling thermostat coupling. Prior to production, all systems underwent a multistep equilibration protocol: 1 ns under *NVT*, followed by 1 ns under *NPT* to allow box-size adjustment, and a final 1 ns under *NVT* conditions.

### 2.6 Studies with Cells

#### Cells and Culture Media

The human A2780 ovarian cancer cells were obtained from Sigma-Aldrich. For the experiments, cells were cultured in RPMI-1640 medium (Gibco, Thermo Fisher Scientific) supplemented with 10% FBS and maintained at 37 °C in a 5% CO_2_ humidified atmosphere.

#### Cellular Viability

For the cellular viability studies, A2780 cells were seeded in 96-well plates (1–2 × 10^4^ cells/200 μL medium) and incubated at 37 °C for 24 h to adhere. Then, the medium was discarded, and cells were incubated with the several types of vanadate solutions in the cell media for 24 h. After incubation, the viability was determined using the MTT assay as previously reported.^130^ Similar cellular viability experiments were carried out with solutions of free organic compounds 4-Me2AmPy and 1-MeIm.

#### Cellular Uptake by ICP-MS

A2780 cells at ∼10^6^ cells/2 mL were incubated for 24 h with 50 μM of ‘V_10_ solution’ or ‘V_1_ solution’, with or without 150 μM of 4-Me2AmPy. To evaluate possible effects due to changes in composition of the samples, similar experiments were carried out first by adding the solutions to the cell media, keeping these for 24 h at 37 °C, and then incubating the A2780 cells with these preparations. After, the medium was discarded, and the cell pellet was collected upon trypsinization. After two washing cycles with cold PBS, the cell pellets were kept and frozen at −80 °C till analyzed. For analysis by ICP-MS, the cell pellets were digested with nitric acid (0.5 mL, 65%) at 100 °C for 12 h. Each solution was diluted in Milli-Q water to 10.00 mL. The total content of V was measured by a Thermo X-Series Quadrupole ICP-MS instrument (Thermo Scientific). Standard solutions were prepared from ICP-MS 71 A (Inorganic Venture) with a final concentration of 5.0% nitric acid. Indium (^115^In) at a concentration of 10 μg/L was used as an internal standard. The V levels for each condition were expressed as nanograms of V/10^6^ cells.

In the experiments where decavanadates and one of the organic cations are simultaneously present, these were carried out using solutions of V_1_ or V_10_, and the organic compound was added separately.

#### pH Measurements with Media

A few experiments were carried out to measure the pH of the cell media in contact with the ‘V_10_ solution’ and the ‘V_1_ solution’ to check its possible change with time. In all cases, a [V]_total_ of 50 μM was used. Three types of experiments were made: (i) measurement of pH of the RPMI-1640 incubation media; (ii) measurement of pH of the RPMI-1640 incubation media after being kept at 37 °C during 24 h; (iii) measurement of pH of the supernatant of the RPMI-1640 incubation media after being in contact with A2780 cells during 24 h at 37 °C.

## 3 Results and Discussion

### 3.1 Structure Description

The crystal structure of new compounds **1**–**3** was established by single-crystal X-ray diffraction. Table 1 presents the corresponding crystal data, and Tables S1 and S2 contain additional crystallographic information. The molecular structures of **1**–**3** are depicted in Figures 1–3, and selected bond distances and angles are presented in Table S1 (Supporting Information). Compound **1**, (H.4-Me2AmPy)_4_[H_2_V_10_O_28_], crystallized in the triclinic system, *P*-1 space group, as bronze prisms (Figure 1). The asymmetric unit presents half decavanadate anion and two H.4-Me2AmPy^+^ cations due to the intersection of the structure by a crystallographic inversion center. The (−4) charge balance of the diprotonated decavanadate anion is further stabilized by intermolecular hydrogen bonds between these four H.4-Me2AmPy^+^ cations and cluster oxygen atoms O3 and O7 (see Table S2 and Figure S4 in the Supporting Information), leading to the general formula (H.4-Me2AmPy)_4_[H_2_V_10_O_28_]. The protonation effect on 4-Me2AmPy mainly results in an increase in the internal C–N_py_–C angle, whereas the bond lengths remain almost unaltered. This angle displays values of 120.7(3)° and 120.5(3)°, for molecules **1** and **2** in the asymmetric unit, respectively, while in 4-Me2AmPy, the angle is 114.70°.

**Figure 1:**
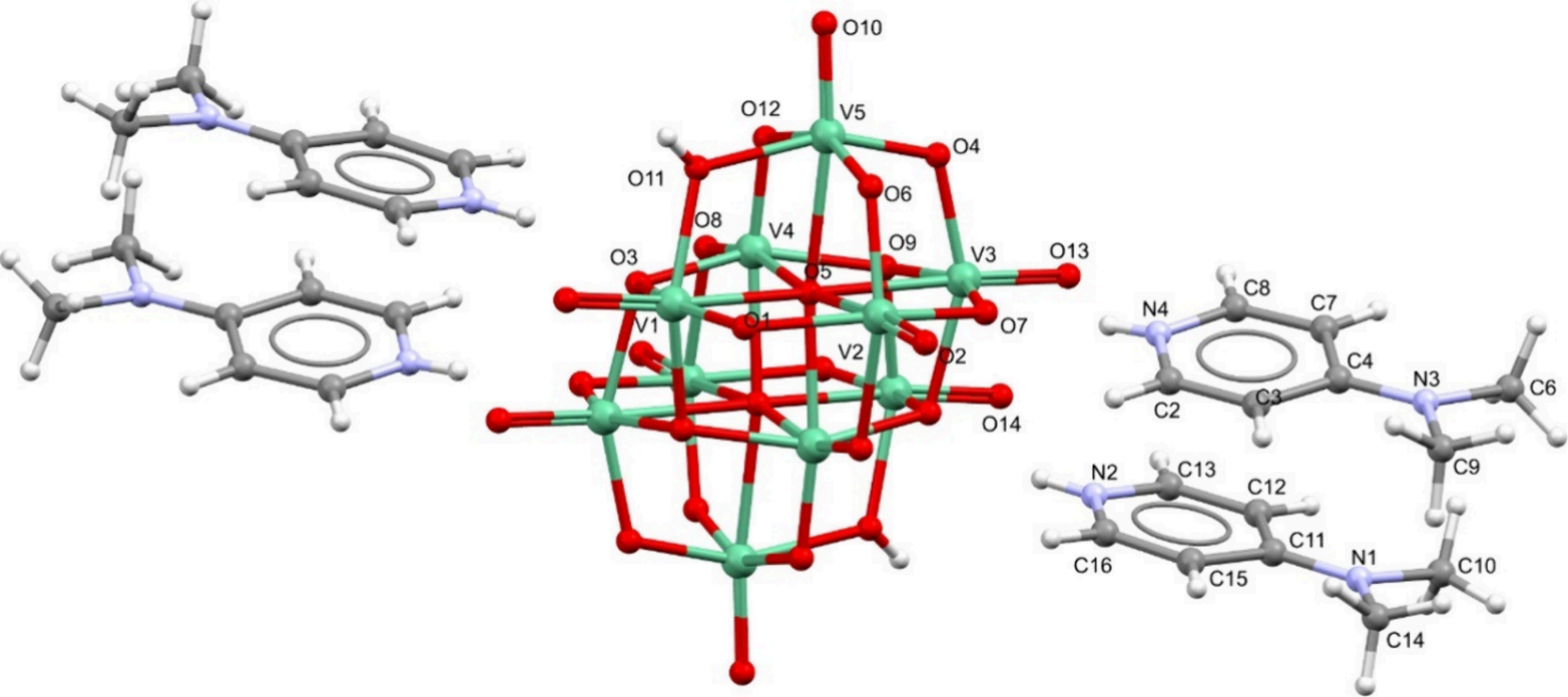
Molecular structure of **1**, (H.4-Me2AmPy)_4_[H_2_V_10_O_28_], with the corresponding labeling scheme. Vanadium atoms are represented in green. The two hydrogen atoms present at the anion were located on atoms O11 and O11#. Figure S4­(C) depicts an Ortep drawing of the molecular structure of **1.**

**Figure 2:**
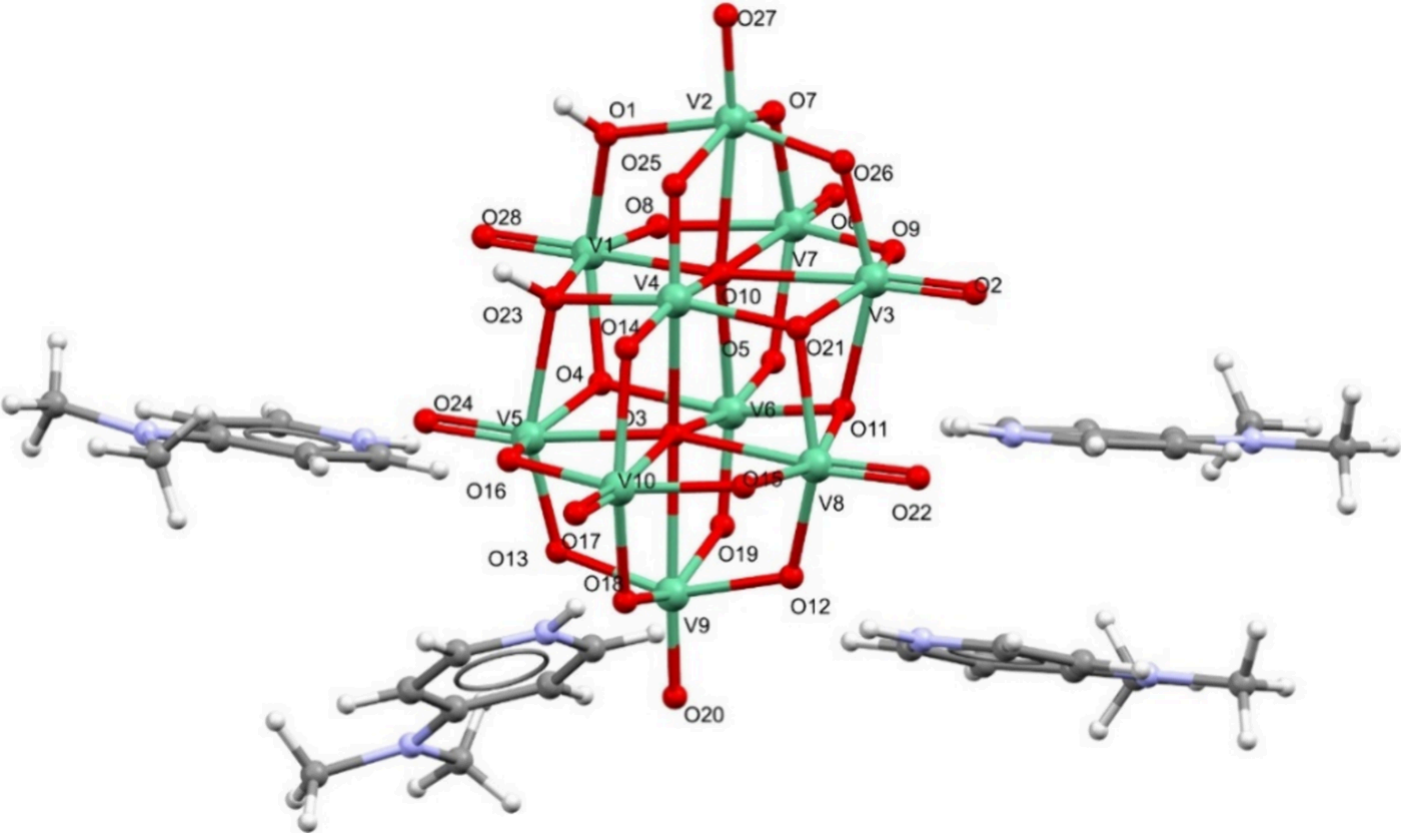
Molecular structure of **2**, (H.4-Me2AmPy)_4_[H_2_V_10_O_28_], with the corresponding labeling scheme. Vanadium atoms are represented in green. The two hydrogen atoms present at the anion were located on atoms O1 and O23. Figure S6­(C) depicts an Ortep drawing of the molecular structure of **2.**

**Figure 3:**
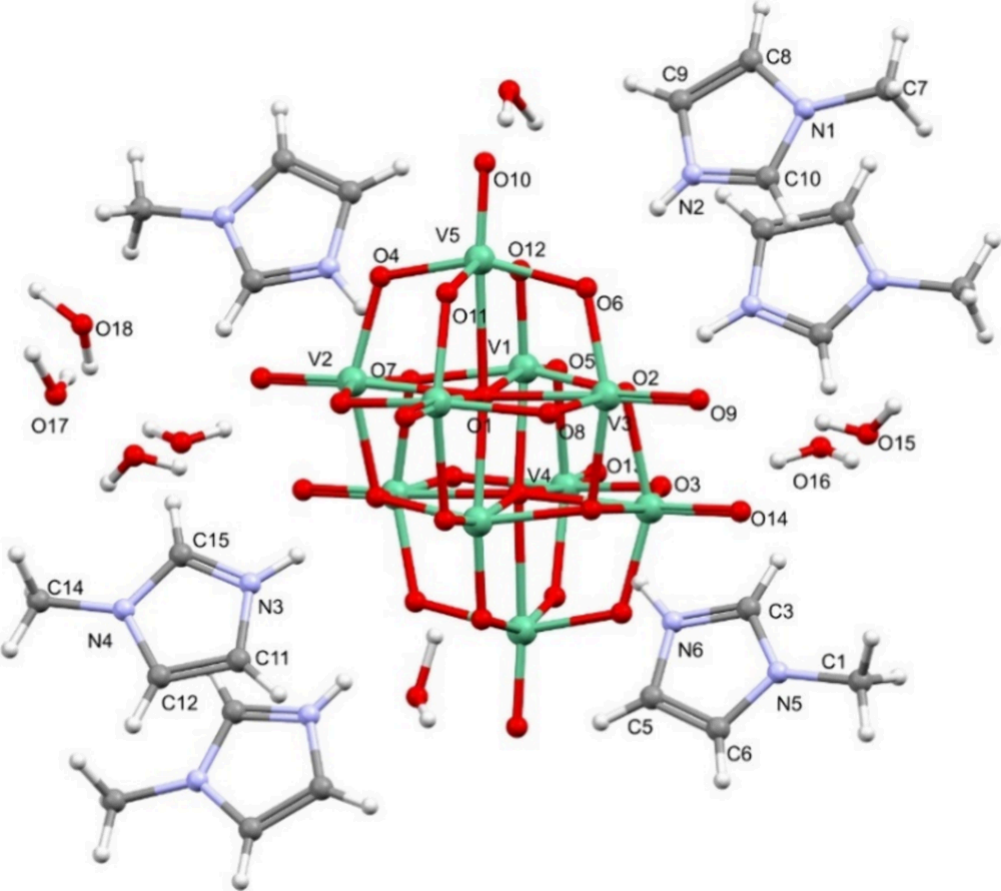
Molecular structure of **3**, (C_4_H_7_N_2_)_6_[V_10_O_28_]·8H_2_O, with the corresponding labeling scheme. Vanadium atoms are depicted in green. Figure S7­(C) depicts an Ortep drawing of the molecular structure of **3**.

The decavanadate anion is composed of three types of VO_6_ octahedra with V–O distances in the range 1.595(2) to 2.3273(19) Å (Table S1). The geometry around each vanadium atom is that of a distorted octahedron, as the O–V–O angles deviate from the theoretical 90 and 180° for *cis* and *trans* O atoms, respectively (Table S1). The two O atoms located inside the cluster, O5 and O5#, are bonded to six V atoms and show the largest distances within the cluster (2.0813(19)–2.3273(19) Å). Oxygen atoms O3, O3#, and O9# are coordinated to three V atoms each, with bond distances between 1.917(2) and 2.063(2) Å. Eight O atoms (O2, O10, O13, O14, O2#, O10#, O13#, and O14#) display terminal positions and coordinate to a single V atom with V=O bond lengths ranging 1.595(2) to 1.619(2) Å, being the shortest within the anion. The remaining O atoms are bridging two vanadium atoms and display bond distances between 1.676(2) and 2.059(2) Å. These bond lengths are in agreement with other reports in the literature.^38,96,131–135^

Hydrogen atoms bonded to decavanadate O atoms were localized on oxygen atoms O11 and O11#. For this purpose, Fourier maps were used, considering simultaneously the residual electron density and the distances within the decavanadate structure. The supramolecular arrangement in **1** is generated by hydrogen bonds of the type N–H···O and O–H···O, combined with parallel offset π–π stacking between displaced H.4-Me2AmPy^+^ rings within 3.727 Å, which is in the accepted range for this type of π–π interaction (Figure S4 and Table S2). *R*_2_^2^(8) motifs can also be observed between two diprotonated decavanadate anions.^133^

When analyzing the batch of crystals obtained for **1**, a second type of morphology was observed. The structure of these yellow prisms was determined, leading to the elucidation of the crystal structure of compound **2** (Figure 2). Selected bond distances are listed in Table S1. This compound, with the general formula (H.4-Me2AmPy)_4_[H_2_V_10_O_28_], crystallized in the monoclinic system, *P*2_1_/*n* space group, corresponding, in fact, to a polymorph of **1**. The superposition of **1** and **2**, shown in Figure S5, clearly shows that, in these polymorphs, the V_10_ clusters are perfectly overlaid, whereas the H.4-Me2AmPy^+^ cations are in different positions of the asymmetric unit. This is related to the intermolecular interactions observed in the two crystal structures. In the case of **2**, five N–H···O and two O–H···O hydrogen bonds are present in the asymmetric unit (Figures S6­(A,B) and Table S2). In compound **1**, as seen above, only two N–H···O and one – H···O can be observed. In the diprotonated V_10_ anion, all the V–O bond lengths are within the range observed for compound **1** (Table S1), with distances of 2.083(3)–2.368(3) Å for μ_6_-O atoms, 1.864(3)–2.1389(8) Å for μ_3_-O atoms, 1.6785(8)–2.0911(9) Å for μ_2_-O atoms, and 1.589(3)–1.621(3) Å for V–O_terminal_ atoms. The two hydrogen atoms present at the anion were located on atoms O1 and O23. In addition, the C–N_py_–C angle in H.4-Me2AmPy^+^ is ca. 120°, being similar to what is observed in the molecular structure of **1**. The π–π stacking between some of the H.4-Me2AmPy^+^ rings correspond to shorter distances in **2** (3.494 Å in **2** (Figure S6), vs. 3.727 Å in **1** (Figure S4)).

Crystals suitable for single-crystal X-ray diffraction were also obtained for compound **3** containing protonated 1-*N*-methylimidazole (1-MeIm) cations, which crystallized in the monoclinic system, *P*2_1_/*n* space group, as yellow prisms. Half-cluster, three protonated 1-*N*-methylimidazoles (H.1-MeIm^+^) and four cocrystallized water molecules are displayed in the asymmetric unit, leading to the general formula (C_4_H_7_N_2_)_6_[V_10_O_28_]·8H_2_O. Its molecular structure is depicted in Figure 3 and selected bond distances are shown in Table S1 (Supporting Information).

In compound **3**, the [V_10_O_28_]^6–^ charge is balanced by six protonated 1-*N*-methylimidazoles. The *N*-methylimidazole NH protons were located on Fourier maps. Their C–N­(H)–C angles are in the range 108.06°–109.08°, being larger than those observed for the nonprotonated derivative.^134^ This is in agreement with what was observed for the H.4-Me2AmPy^+^ derivatives in compounds **1** and **2**. The V_10_ anion shows features similar to those in **1** and **2**, in terms of bond distances. In fact, in the μ_6_-O atom, distances vary between 2.0869(17) and 2.3390(18) Å, in μ_3_-O atoms, they range from 1.9251(17) to 2.0413(19) Å, while for μ_2_-O atoms, they are between 1.6822(18) and 2.0651(18) Å, and finally, the V–O_terminal_ atoms have bond lengths in between 1.597(2) and 1.6172(18) Å. The crystal structure is further stabilized by the presence of eight water molecules and their consequent −H···O hydrogen bonds (Table S2 and Figure S7­(A)). Furthermore, the compound is further stabilized by the establishment of N–H···O hydrogen bonds and by the formation of parallel offset π–π stacking between displaced 1-MeIm rings with distances of 3.331 and 3.617 Å (Figure S7­(B)). These intermolecular interactions give rise to a supramolecular arrangement where the V_10_ anions are located at the vertices and center of the unit cell and the *N*-methylimidazole cations and water molecules are encircling the central [V_10_O_28_] unit (Figure S8).

Bond valence sum (BVS) calculations (Table S3), calculated using the Brown and Altermatt's methodology,^136^ revealed that all V atoms have valence sums close to 5, confirming, as expected, the formulation of the cluster anion as [H_4_V^V^_10_O_28_]^4–^. This analysis also allowed us to locate the two protonation sites of the V_10_ clusters in compound **1** on O11 (valence: 1.331); because of the 2-fold cluster symmetry, the single protonation on O1 results in two protonation sites when the crystallographic symmetry elements are applied, so that the complete cluster is 2-fold protonated. In compound **2**, the O atoms of the V_10_ anion, O1 (valence: 1.331) and O23 (valence: 1.309), show comparatively low bond valence sums, which are reasonable values for protonated O atoms.

There were previous reports of the SC-XRD characterization of V_10_ anions with 4-dimethylaminopyridinium. Two of them contain the same organic cations: (4-Me2AmPy)_6_[V_10_O_28_], differing in the lattice water content.^137^ The (4-Me2AmPy)_4_(NH_4_)_2_[V_10_O_28_]·8H_2_O was also characterized,^138^ as well as (4-Me2AmPy)_4_[V_10_O_28_]·5H_2_O.^139^

More recently, crystals of (4-Me2AmPy)_4_[H_2_V_10_O_28_]·2DMSO were obtained.^139^ The crystal structures of compound **1** and 4-dimethylaminopyridinium decavanadate (CSD refcode GIVHEC) were reported and they are closely related.^140^ In fact, GIVHEC is a DMSO solvate of compound **1**, which is reported herein. Both compounds crystallize in the triclinic system (*P*-1 space group), and the differences in the unit cell parameters are primarily due to the presence of the DMSO solvate. Additionally, π–π stacking interactions exist between the aromatic rings of the 4-Me2AmPy cation, with a centroid-to-centroid separation of approximately 4.4 Å in GIVHEC, whereas in compound **1**, this value is 3.7 Å. Conversely, when observing the structures of the derivatives containing imidazole cations (compound **3** vs GIVHAY^140^), it is possible to comment that, in this case, they are not closely related. While GIVHAY displays a diprotonated V_10_ anion stabilized by four imidazole cations, compound **3** shows a nonprotonated decavanadate anion surrounded by six imidazolium cations and four crystallization water molecules.

### 3.2 IR Spectra of Compounds **1**–**3**

The infrared spectra of compounds **1**–**3** are depicted in Figures S9­(A,B). The characteristic peaks for the skeletal vibration of [H_*n*_V_10_O_28_]^(6–*n*)–^ decavanadates in the region between 500 cm^–1^ to 1000 cm^–1^ are as expected for POV compounds that have almost the same basic framework. The characteristic strong bands associated with the terminal V=O stretching mode appear at around 960 cm^–1^ for **1**–**3**. The bridging antisymmetric vibrations of V–O–V probably correspond to the bands in the range 730 and 840 cm^–1^, and the symmetric vibrations are probably in the range 515 and 600 cm^–1^, in agreement with FTIR spectra for related compounds.^26,46,101,102,104,101,102,141–145^ The bands at ν = 2660–2940 cm^–1^ can be associated with C–H stretching and the two bands in the region ν = 3550–3000 cm^–1^ are attributed to stretching of hydrogen-bonded N–H moieties.^146^ In the same region, the IR spectrum of compound **3** also confirms the presence of water molecules and of 1-methylimidazolium^+^. In fact, the broad absorptions between about 3600 and 3450 cm^–1^ are mainly caused by the O–H stretching vibration of lattice water molecules, and those in the range of ca. 3300 and 3100 cm^–1^ are mostly due to symmetric and asymmetric N–H modes of the organic cations. We remark that, in compounds **1** and **2**, there are no relevant bands in the 3600–3450 cm^–1^ range, further indicating the interesting observation that these decavanadate compounds have no associated water molecules in their crystal structure. The O–H and N–H bending vibrations are observed at 1646 and 1578 cm^–1^, the CH_2_ bending mode is at 1458 cm^–1^, while the C–H stretching modes of CH_3_ are between 2536 and 2955 cm^–1^. The absorption peaks in the range of 1463–1180 cm^–1^ correspond to the vibration of imidazole moieties.^6^

### 3.3 Hirshfeld Surface Analysis

Concerning the crystal packing, the several existing noncovalent interactions are important aspects to be examined because besides allowing further understanding the details about the arrangement of molecules in the crystalline material, they give clues to their potential for interaction with biologically relevant molecules. To evaluate noncovalent interactions, the Hirshfeld surface (HS) analysis is often used, and these studies may be carried out using available software such as the Crystal Explorer (3.1). All of the available space around molecules is somewhat taken into account.^147–151^ The HS surface shape is a means of globally describing intermolecular interactions in the compound, evaluating the interplay between the atoms present and their intermolecular contact distances. Notably, besides reflecting the interactions between the molecules present in the crystal, it is also associated with interactions between atoms in individual molecules.

The HS can be made by using various characteristics such as *d*_norm_ (normalized distance), shape index, and so forth. Each surface gives information on the noncovalent interactions. The HS of a molecule traced on the *d*_norm_ contains three colors, red, blue, and white, which indicate interatomic contacts where the distance between the atoms is less than or equal to the sum of the van der Waals radii of the atoms concerned, respectively. The blue areas of the HS show that the space between atoms is larger than the sum of the van der Waals radii of the atoms concerned. It can be said that red, white, and blue on the HS indicate strong, comparatively low, and quite low and nonrelevant intermolecular interactions, respectively. Not only H-bonding interactions can be displayed by tracing HS, but we can examine weak interactions of π···π stacking type. To visualize these interactions, the HS is plotted on a shape index. π···π stacking interactions are indicated in the HS by the presence of consecutive triangle regions of red and blue around the aromatic rings.

For the three compounds, successive triangular regions of red and blue are present on the HS around the aromatic cycles, indicating that these π···π stacking interactions are relevant (Figure 4).

**Figure 4:**
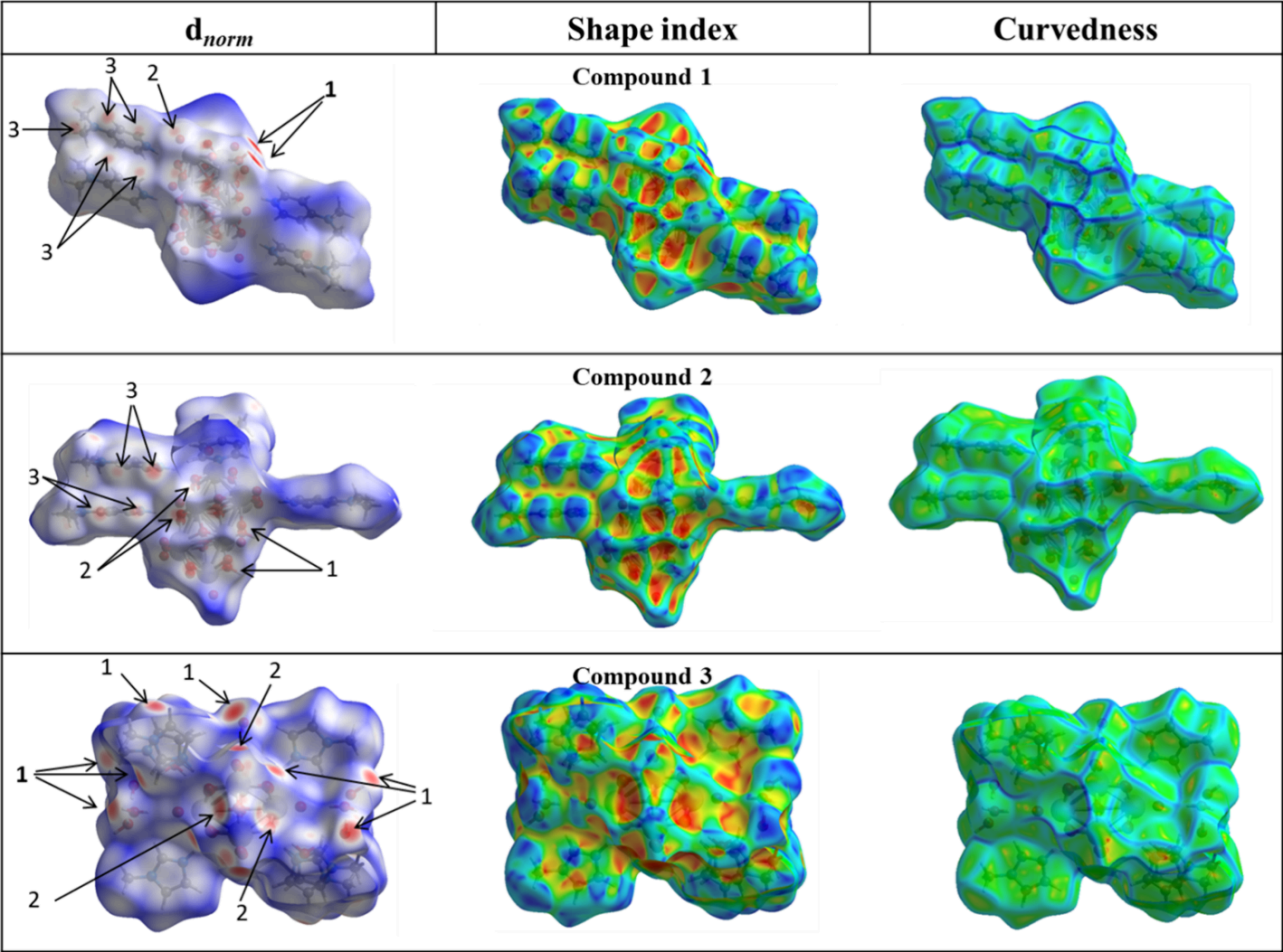
HS plotted considering the *d*_*norm*_ values for compound **1** (in the range −0.688 to 2.806 Å), compound **2** (in the range −0.684 to 1.815 Å), and compound **3** (in the range −0.634 to 1.157 Å). HS is also plotted considering characteristics designated as shape index and curvedness for three compounds.

Thus, to better visualize hydrogen bonds and π-stacking interactions in the context of the supramolecular self-assembly of the compounds, the Hirshfeld surfaces and the associated 2D fingerprint plots were calculated. Figure 4 depicts surfaces mapped over the *d*_*norm*_, shape index, and curvedness properties for compounds **1–3**. The red regions labeled 1 in the *d*_*norm*_ maps are attributed to O–H···O hydrogen bonds involving the acceptor O atoms between two decavanadate polyanions (in compounds **1** and **2**) and two water molecules and between polyanion and water molecules (in compound **3**), belonging to two different molecules (symmetry codes in Table S2). In addition, the molecules linked by N–H···O hydrogen bonds are visible as deep-red spots labeled 2 in the *d*_*norm*_ surfaces. In addition to the nonclassic hydrogen bonds, C···C contacts are clearly visible as a pair of tow deep-red labeled 3. The spots indicate the formation of a centrosymmetric stacking pair involving H.4-Me2AmPy^+^ for compounds **1** and **2.**

On the other hand, there is a touching complementary pair of triangles for the shape index surface and large and green flat regions for the curvedness surface (Figure 4), which indicate that relevant π-stacking interactions (intercentroid distances shorter than 3.8 Å) are present in the crystal packing for the referred structures. The contact of the ···-H···O atom represents the stronger hydrogen bond interaction in the crystal, as reflected by the geometric parameters shown in Table S2.

Fingerprint plots for the main intermolecular contacts of compounds **1–3** are shown in Figure S10. The shortest contacts correspond to very close H···H contacts for the three compounds. These contacts are not visible on the Hirshfeld surface because their distances are longer than the sum of the van der Waals radii. The pairs of narrow pointed spikes labeled 1 and 2 around (*d_e_* + *d_i_*) of 2.5 and 2.6 Å show the presence of O···H and N···H contacts, respectively, where *d_e_* and *d_i_* are the distances from the point to the nearest nucleus external or internal to the surface, respectively.

The relative contributions of the intermolecular interactions bonds and contacts to the Hirshfeld surface area for three compounds are shown as a histogram in Figure S11. The interactions of the O···H/H···O bond and H···H contacts for three compounds have a major contribution to the crystal packing, and the C···C (π··· π) interactions comprise 1.2%, 3.3%, and 2.2% of total Hirshfeld surface area of compounds **1–3**, respectively, whereas N···H/H···N, O···O, C···H/H···C, N···C/C···N, and N···C/C···N contacts have their significant contribution to the total area of the surface. The remaining contacts are negligible.

### 3.4 Studies in Solution

#### UV–vis Spectra

Figure 5 depicts UV–vis spectra of solutions of vanadate­(V) with [V]_total_ = ∼ 4.99 mM at different pH values. It is clear that between pH 3 and 4.5 the spectra almost coincide, with λ_max_ at 220, ∼240, and ∼350 nm (possibly also at ∼400 nm). Small differences are noticed at pH = 5.0 and quite distinct spectra are obtained at pH = 2.5. This is consistent with the speciation diagram predicted for oxidovanadate­(V) solutions in these conditions (see Figure S12), where more than 95% of V^V^ is in the form of H_*n*_V_10_O_28_^–6+*n*^ species, except at pH = 5 where although ca. 10% of V is not in the form of V_10_ anions, their decomposition is rather slow at this pH. The UV–vis spectra of V_10_ anions thus do not change much with their protonation.^147^ Therefore, whenever distinct spectra are obtained for solutions containing only simple salts of oxidovanadate­(V) in the pH range 2–9, that is because V_10_ anions no longer predominate in solution.

**Figure 5:**
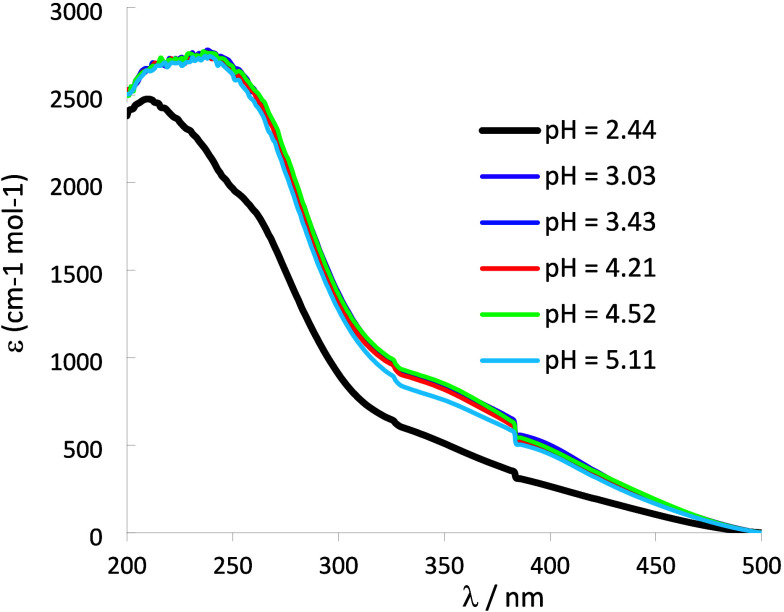
UV–vis spectra of solutions of oxidovanadate­(V) with [V]_total_ = 4.99 mM, prepared from 1:10 dilutions (each in distinct 10.00 mL volumetric flaks, adjustment of pH and final set up of the total volume) of a ‘V_10_ solution’ of pH = 4.0 with [V]_total_ = ∼49.9 mM. Each spectrum was recorded after ca. 2 h of preparation of the corresponding solution with a cell of 2 mm path length. Absorption maxima are at ∼220 and ∼240 nm and a shoulder band at 345–350 nm.

Figure 6 depicts UV–vis spectra of solutions of oxidovanadate­(V) with [V]_total_ ranging from 10 to 80 μM in Hepes buffer at pH = 7.0. It is very clear that immediately after their preparation, the UV–vis spectra of the solutions at pH = 7.0 are already quite different from those shown in Figure 5, and differences are more noticeable as [V]_total_ decreases. Figure S13 depicts speciation diagrams of solutions of oxidovanadate­(V) at pH = 7.2 in the ranges 10 to 100 μM (Figure S13­(A)) and 10 to 1000 μM (Figure S13­(B)). We emphasize that, from the thermodynamic point of view, at this pH and V^V^ concentration range, the V_10_ anions should not exist, V_1_ being the most relevant species followed by V_2_ (max. 8%). Notwithstanding, the UV–vis spectra recorded immediately after their preparation (∼0 h, Figure 6(A)) depict some slight resemblance with those of V_10_ anions, indicating that these UV–vis spectra do not correspond to equilibrium conditions. Upon keeping these solutions at room temperature (RT) for ∼24 h, hydrolysis of V_10_ anions is much more extensive and the UV–vis spectra measured (Figure 6(B)) totally differ from those shown in Figure 5; thus, the compositions of the solutions are much closer to equilibrium conditions. The spectra depicted in Figure 6(B)) also resemble those corresponding to V_1_ and V_2_ species reported by Boreen et al.^152^

**Figure 6:**
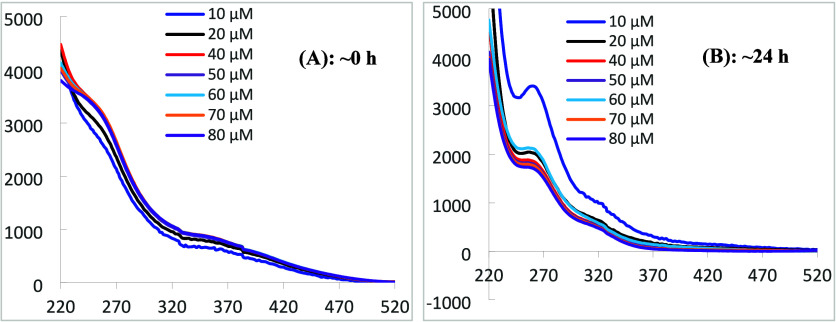
UV–vis spectra of solutions of oxidovanadate­(V) with [V]_total_ concentrations ranging from 10 to 80 μM in Hepes buffer of pH = 7.02. Each solution was prepared from dilutions of a solution with [V]_total_ = 4.99 mM in water, prepared from a 1:10 dilution of the ‘V_10_ solution’ of pH = 4.0 with [V]_total_ = ∼49.9 mM (see experimental section). The initial dilutions were made shortly before the preparation of the solutions used to record the UV–vis spectra (*t* = 0 h). Each spectrum was recorded (A) almost immediately after preparation of the corresponding solution with a cell of 10 mm path length and (B) the same solutions after being kept ∼24 h at room temperature. During the 24 h interval, the pH decreased slightly (around 0.02 pH units), except for the experiments with [V]_total_ of 70 and 80 μM, where an increase of ca. 0.01 pH units was observed.

**Figure 7:**
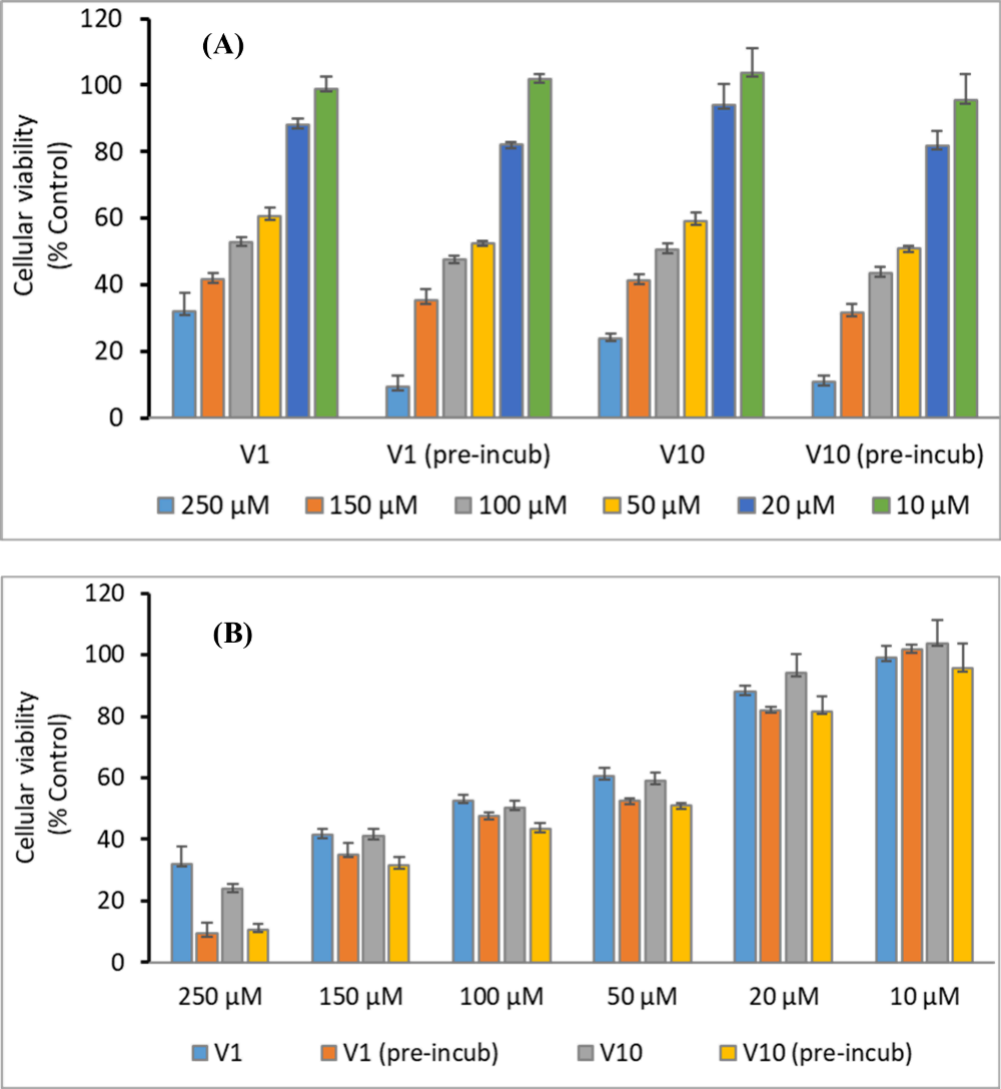
Cellular viability as % control (untreated cells) of decavanadate (V_10_) and vanadate (V_1_) solutions with and without previous incubation (pre-incub) in cell medium for 24 h, at 37 °C before addition to the A2780 cells. Results are mean ± SD of two independent experiments done with four replicates. (A) The data for each type of solution as a function of [V]_total_ and (B) the results are displayed for each [V]_total_ tested. Note that the total vanadium concentration for each set of columns is approximately equal. For example, for the set of columns specifying 150 μM, for the gray and yellow columns, this means that the amount of decavanadate initially added corresponds to a concentration of 15.0 μM.

#### 3.4.2 ^51^NMR Spectra of Oxidovanadate­(V) Solutions

^51^V NMR spectra of solutions of oxidovanadium­(V) salts and complexes have been extensively studied, both experimentally^74,153–155^ and by theoretical calculations.^156–158^ Namely ^51^V NMR was used to determine which are the main species present in solution and calculate the corresponding stability constants.^153–155^

In this work, the ^51^V NMR spectra were measured either to confirm that stock solutions of decavanadates at pH = 4.0 only contained V_10_ species (e.g., Figure S1) or that stock V_1_ solutions did not contain decavanadate species (e.g., Figure S3); globally, one of the main objectives of the measurement of ^51^V NMR spectra was to disclose the type of V-containing species present in solution, for example at different pH values, in incubation media of cells and in the cell media after contact with cells.

The V_10_ anions contain three distinct types of V atoms according to their position in the polyanion structure, which are often labeled as V10A, V10B, and V10C. In this work, we labeled as V10A the 6-coordinate nonoxido V atoms that are buried inside the V_10_ polyanion; these are the vanadium atoms less sensitive to modifications in the media conditions, such as changes of pH. The external V atoms containing a V=O group are labeled V10B and V10C; these atoms are close to the surface and accessible to species of the media and show the greatest sensitivity to modifications of the pH of the solution.

The ^51^V NMR chemical shifts of these V atoms (δ_V_) depend on pH due to the change in protonation state of the V_10_ anions. In the ^51^V NMR spectrum of one of the stock solutions designated by ‘V_10_ solution’ at pH = 4.0 (Figure S1), only peaks due to V_10_ anions are detected. Upon addition of a base up to pH = 5.3 to these solutions and recording the ^51^V NMR spectrum shortly afterward (Figure S2), as expected from the species distribution diagram (e.g., Figure S10), decavanadate species continue being the predominant ones, but V_1_, V_2_, and V_4_ are also clearly visible. Below, we discuss the ^51^V NMR spectra measured with RPMI-1640 incubation media before and after contact with A2780 cells.

### 3.5 Computational Study

We carried out classical MD simulations and quantum mechanical DFT calculations, which tackle combinations of both cations with V_1_, V_2_, V_4_, and V_10_ compounds to explore their mutual influence and the resulting physicochemical properties in aqueous solution. First, the DFT-optimized structures for the vanadate compounds as well as for H.4-Me2AmPy^+^ and H.1-MeIm^+^, were obtained and then used in the MD simulations. Because of the ionic nature of the components, the MD trajectories exhibit a certain extent of ion pairing, with the degree varying depending on the cation and the specific vanadate species involved. The combination of H.4-Me2AmPy^+^ and V_10_ averages to *N* = 2.26 cations aggregated to every V_10_ unit, with a mean distance between their respective centers of mass of 7.38 Å (as shown in Figure S14). In contrast, the simulation with H.1-MeIm^+^/V_10_ reveals that, on average, fewer than one cation is ion-paired per POV unit. This clearly indicates a much higher affinity of H.4-Me2AmPy^+^ for V_10_ compared to that of the imidazolium-based cation under these conditions. This particular trend is also observed if V_4_ is considered, with 2.52 H.4-Me_2_AmPy^+^ cations and 0.70 H.1-MeIm^+^ cations aggregated. Two more simulations, with H.4-Me2AmPy^+^/V_2_ and H.1-MeIm^+^/V_2_, still show more different ion pairing: 2.62 and 0.30 cations per POV unit. Only when V_1_ is considered, these numbers decrease to less than 0.2 in both cases, indicating that the mononuclear vanadate does not present affinity for the cations in aqueous solution (Table S4). Thus, it can be inferred that the physicochemical properties of V_2_, V_4_, and V_10_ will be better described if the effects of H.4-Me2AmPy^+^ are included in the calculations.

The MD results are valuable not only for describing the interactions between species from a purely structural perspective but also for providing superstructures (aggregates) that can be further analyzed at the quantum mechanical level. As will be discussed below, not considering both species combined can lead to inaccurate results. Therefore, we have taken five representative superstructures of the H.4-Me2AmPy^+^/V_10_ system and calculated their protonation and reduction energies in water, allowing comparison with the bare V_10_ molecule. Scheme 1 presents the energies obtained (in eV) for pure protonation, pure reduction, and proton-coupled electron transfer (PCET) for the V_10_ molecule and its derivatives. The values were obtained without (in black) and with (in red) bound cations at positions taken from the MD trajectory to stress the effect of ion pairing.

**Scheme 1:**
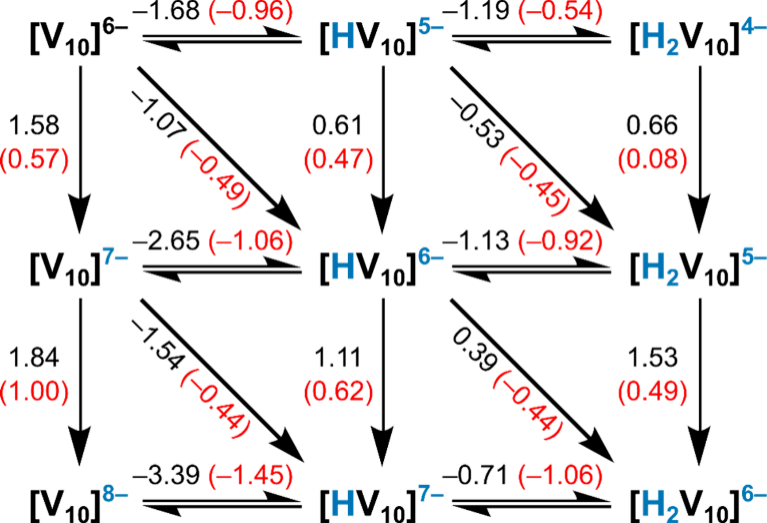
Energies (in eV) for Pure Protonation, Pure Reduction, and PCET Processes for the V_10_ Anion and Its Derivatives^a^

a The V_10_O_28_ entity is abbreviated as V_10_.

Not taking ion pairing into account, the V_10_ anion has a strong tendency to protonate as the reaction energies in the first line of Scheme 1 suggest (−1.68 eV to gain the first proton and additional −1.18 eV to gain the second proton). Such large negative values assume an abundance of protons in the medium (low pH). In line with this, Figure S12 shows that at any pH value, the nonprotonated form is the least abundant decavanadate-based species, and that the biprotonated species prevails over the monoprotonated one in acidic media. The energy involved in the third protonation, not shown in Scheme 1, [H_2_V_10_]^4–^ + H^+^ → [H_3_V_10_]^3–^ (or [H_2_V_10_O_28_]^4–^ + H+ → [H_3_V_10_O_28_]^3–^) is −0.43 eV, substantially lower than the previous two, as expected. This also correlates with a residual relative abundance of the triprotonated form if compared with H_2_V_10_ ([H_2_V_10_O_28_]^4–^).

The protonation processes are, in general, less favored when ion pairing is considered, whereas pure reductions are more favorable if cations are taken into account. Looking at PCETs, it depends on the initial state that the process is more ([HV_10_ 1e]^6–^ + e^–^ + H^+^ → [H_2_V_10_ 2e]^6–^), less ([V_10_]^6–^ + e^–^ + H^+^ → [HV_10_ 1e]^6–^), or similarly ([HV_10_]^5–^ + e^–^ + H^+^ → [H_2_V_10_ 1e]^5–^) favored for the aggregate species (note in this and in the previous paragraph we are using V_10_ as an abbreviation of V_10_O_28_). The 1e and 2e labels indicate that the system has gained one or two electrons from the fully oxidized form, [V^V^_10_]^6–^, these electrons being partially or fully delocalized. The energies shown indicate different stabilities for the V_10_ derivatives and, if some reducing agent is present, the probable prevalence of the [HV_10_]^6–^, [H_2_V_10_]^6–^, or [H_2_V_10_]^5–^ species over V_10_. Another parameter linked to stability/reactivity is molecular orbital (MO) energies. For the V_10_ species, the highest occupied MO (HOMO) and the lowest unoccupied MO (LUMO) are found at −5.93 and −2.04 eV, respectively, if no aggregated cations are considered (see Figure S14). The same two orbitals have energies of – 6.18 and – 2.34 eV in the ion-paired superstructure, a stabilization that explains the more favorable reduction process [V_10_]^6–^ + e^–^ → [V_10_]^7–^ shown above, and the less favored protonation [V_10_]^6–^ + H^+^ → [HV_10_]^5–^. These trends may be relevant to the biological effects of V_10_ species in the presence or absence of cations.

### 3.6 Cytotoxic Effect on A2780 Ovarian Cancer Cells

The cytotoxic activity was evaluated against A2780 ovarian cancer cells at total oxidovanadate­(V) concentrations in the range 50–300 μM in cell medium, starting from stock ‘V_10_ solutions’ and ‘V_1_ solutions’. Note that in this context, ‘V_10_ solutions’ correspond to those freshly prepared from a concentrated decavanadate solution at pH ≈ 4 (only containing V_10_ ions) and ‘V_1_ solutions’ correspond to V^V^ solutions not containing decavanadates (see experimental for the procedure used). The cytotoxic assays were carried out with fresh dilutions of these solutions in RPMI-1640 which were (i) either immediately added to cells or (ii) after keeping them 24 h in contact with the RPMI-1640 medium at 37 °C.

The results depicted in Figure 7(A) show a dose-dependent decrease of the cellular viability similar for both vanadium forms. The estimated IC_50_ values for the ‘V_1_ solutions’ are 111.8 ± 12.6 μM (without preincubation) and 73.4 ± 12.5 μM (with preincubation), and for the ‘V_10_ solutions’: 103.7 ± 18.3 μM (without preincubation) and 64.8 ± 10.3 μM (with preincubation). Note that these IC_50_ numbers correspond to [V]_total_ values; because the composition of the solutions vary with [V]_total_, the IC_50_ values do not strictly correspond to IC_50_ values of monovanadates­(V) or decavanadates. We highlight that in these experiments with A2780 cells, the IC_50_ values determined after 24 h incubation of cells do not significantly differ for the ‘V_10_ solutions’ and for the ‘V_1_ solutions’, this being a relevant observation. This may be explained assuming that at pH 7–8, the decavanadates hydrolyze rather fast, and for identical [V]_total_ values, the cytotoxicity will be the same. Therefore, any discussion of the biological effects observed with this type of compounds should be carefully made, not assuming that the initial and final composition of the samples are equal, and not making straightforward conclusions from the simple observations made.

Moreover, we are presently not able to explain why in the experiments with 24 h of preincubation of the V^V^ solutions with the RPMI-1640 media, the determined IC_50_ values are significantly lower. We also highlight that, as Figure 7(B) clearly demonstrates, besides the information associated with IC_50_ values, for each [V]_total_ values tested, the cellular viabilities do not significantly differ for either fresh or aged ‘V_10_ solutions’ and ‘V_1_ solutions’.

The cytotoxic activity was also evaluated in the presence of the organic cations of 4-dimethylaminopyridine and 1-methylimidazole at concentration ratios three times those of the vanadates (Figure 8 and Figure S15), with or without 24 h previous incubation in the cell medium at 37 °C; these results were also obtained with A2780 cells but in independent experiments from those of Figure 7. The results presented in Figure 8 show the cellular viability of the cells upon treatment with ‘V_10_ solution’ (200 and 300 μM), 4-Me2AmP and V_10_+4-Me_2_AmPy (600 and 900 μM), with 24 h previous incubation of the compounds with the cell media before their addition to the cells; Figure S15 shows the cellular viability of the cells upon treatment with ‘V_10_ solution’ (50 and 100 μM), 4-Me2AmPy and V_10_+4-Me2AmPy (150 and 300 μM), with and without 24 h previous incubation of these solutions with the cell media. One of the relevant conclusions of these experiments (Figure S15) is that the cellular viability does not significantly differ for fresh and aged solutions of these compounds. As can be concluded from Figure 8 and Figure S15, although compounds 4-Me2AmPy and 1-MeIm are almost not cytotoxic, when mixed with ‘V_1_ solutions’ or ‘V_10_ solutions’, they somewhat further affect the cell viability. In particular, when 1-MeIm is added to the vanadate solutions, the cell viability may be significantly reduced; notably, while 1-MeIm shows no relevant cytotoxicity, in the presence of V_10_ the viability of A2780 cells decreases drastically.

**Figure 8:**
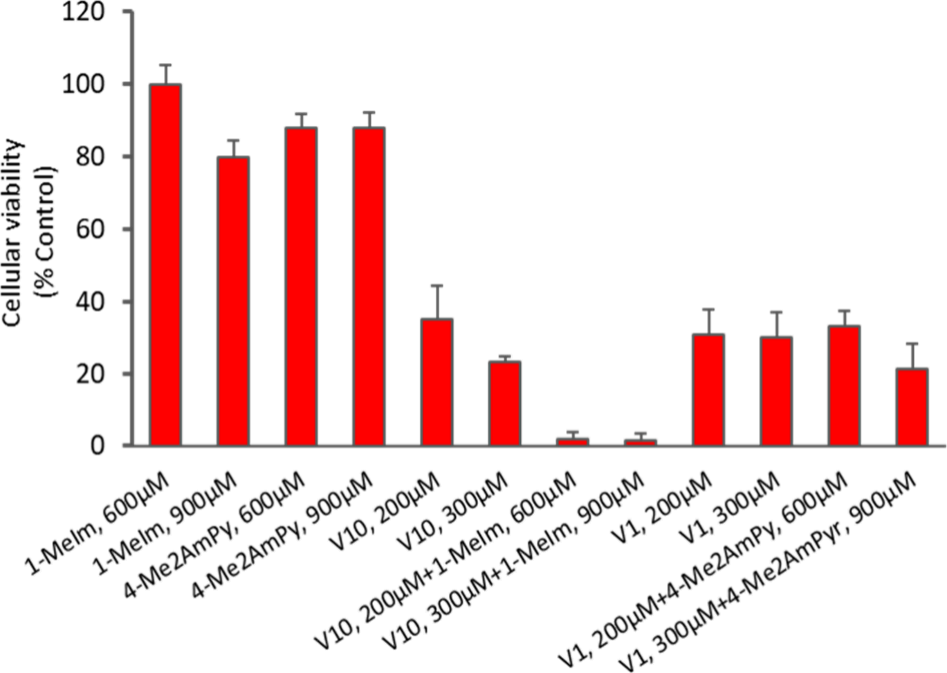
Cellular viability as % control (untreated cells) of V_10_ and V_1_ with previous incubation with the organic cations in cell medium for 24 h, at 37 °C before addition to the A2780 cells. Results are mean ± SD of two independent experiments done with four replicates. Note that the total vanadium concentration for each set of columns is specified. For example, for the set of columns specifying 200 μM, this means that for [V_1_] = 200.0 μM, the amount of decavanadate initially added corresponds to [V_10_] = 20.0 μM, the [V]_total_ being 200.0 μM in both cases.

Globally, the cytotoxicity data emphasize that, regarding the nature/identity of the V-species responsible for the biological effects observed, no straightforward conclusions can be taken without carrying out experiments with and without also testing the organic counterions present, either in the presence or absence of the vanadium compounds. Concerning the effects of decavanadates, previous incubation with cell medium for an adequate period should always also be assayed. Speciation of vanadium species in cell incubation media is also required for the identification of the relevant one responsible for the biological action, as well as the possible interference of the counterions either as promoters of interaction with biological targets or as relevant for redox processes. Even carefully carrying out all these experiments, because several V-containing species are normally simultaneously present and the composition of the solution varies with time and with cell media used, the identification of the species responsible for the biological effects observed and/or determination of mechanism of action probably will remain elusive and doubful.

To the best of our knowledge, only one study was reported on the antitumor activity of decavanadate compounds with ovarian cell lines. The study was carried out with Na_4_Co­(H_2_O)_6_[V_10_O_28_]·18H_2_O (designated as CoV_10_) against SK-OV-3, where an IC_50_ of 0.32 μg/mL was determined.^159^ In this case, a second metal ion is present, Co^2+^, and the ovarian cancer cell line is different, so we do not make any comparison of activities between our compounds and CoV_10_. Moreover, as discussed above, as the composition of decavanadate solutions change with their concentration and with time, we also do not make comparison with anticancer activities reported for other types of cell lines with other decavanadate compounds.

### 3.7 Cellular Uptake Studies by ICP-MS

ICP-MS analysis may be used as a highly sensitive technique to determine the levels of V in the A2780 cell pellets.^69,70,72,160^ Figure 9 depicts graphically the results obtained for the amount of V found in the collected cells after incubation with the vanadate solutions and with the similar solutions also containing 4-Me2AmPy, with and without previous preincubation of the compounds with the RPMI-1640 cell media. As can be observed, the amount of V uptaken by the cells without preincubation of the compounds with cell media is similar for each experimental condition i.e., 7.0, 6.9, 6.9, and 5.8 ng V/10^6^ cells for V_10_, V_1_, V_10_ + 4-Me2AmPy, and V_1_ + 4-Me2AmPy, respectively. Therefore, adding a ‘V_10_ solution’, which initially contains decavanadates, or a ‘V_1_ solution’, which does not contain V_10_ anions, did not have significant impact in the amounts of vanadium uptaken by A2780 cells.

**Figure 9:**
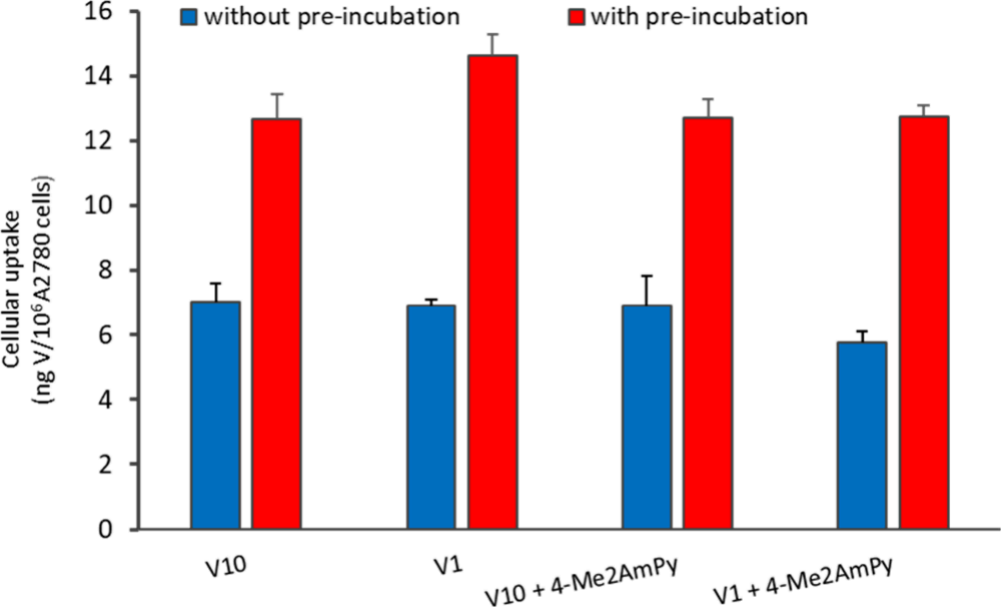
Cellular uptake of vanadium in A2780 cell pellets after treatment with either the ‘solutions of V_10_’ or the ‘solutions of V_1_’, measured by ICP-MS as ng V/10^6^ A2780 cells after 24 h incubation for each experimental condition. Cells were incubated with the ‘V_10_ solution’ and the ‘V_1_ solution’ at 50 μM total vanadium concentration with and without the presence of 4-Me2AmPy assayed at 150 μM. Note that the total vanadium concentration corresponding to the experiments reported in all columns is approximately equal.

Remarkably, in the experiments with preincubation of the V^V^ solutions with the RPMI-1640 media during 24 h, the amount of V found in the cell pellets is similar for all conditions, but present significantly higher uptake values when compared with the former conditions, i.e., 12.7, 14.6, 12.7, and 12.8 ng V/10^6^ cells, respectively for V_10_, V_1_, V_10_ + 4-Me2AmPy, and V_1_ + 4-Me2AmPy. This noteworthy result, i.e. a much higher vanadium uptake in all experiments where preincubation with cell media was done, was quite unexpected and is discussed below.

Several studies addressing the vanadium uptake by erythrocytes were previously reported.^73,161,162^ It is known that monovanadates may be transported through the phosphate anion channels, but this does not allow a straightforward explanation of the data in Figure 9. Once taken up, V^V^ is reduced in intact erythrocytes by intracellular glutathione (GSH) which is depleted from the cytosol. In the intracellular environment of most mammalian cells, enough amounts of GSH and reductase enzymes are present, so that almost all vanadium will be present as V^IV^, part of it bound to glutathione.^161–164^ The uptake of vanadium and the kinetics of the processes were studied by Heinz et al.^162^ They reported that vanadium influx, efflux, and accumulation in erythrocytes (namely involving the V^V^ → V^IV^ reduction), are reasonably fast processes and depend on several factors, such as the extracellular concentration of V^V^, the composition of the cell incubation media, the O_2_ tension, and the pH of the cell media.

Of all factors that may affect the V uptake, contributing to a reasonable explanation of the different data of Figure 9 for experiments with and without preincubation of the vanadate solutions with the cell media, is the possible change of pH during the 24 h of preincubation and the contact with cells. For example, Heinz et al.,^162^ with red blood cells, reported for the vanadium uptake at pH = 7.2 the double of that determined at pH 7.8, all other conditions being similar.

In Table 2, we present measurement of pH values of RPMI-1640 incubation media under several distinct conditions. As the main agents that may act as buffers, this medium contains sodium bicarbonate (23.8 mM) and Na_2_HPO_4_ (5.63 mM). However, the p*K*_a1_ of H_2_CO_3_ is ∼ 6.35, so it is not a suitable buffer for pH ∼ 8.0, the initial pH of the RPMI-1640 media, while phosphate is more adequate for this purpose (p*K*_a2_ ∼ 7.2). Nevertheless, from Table 2 we may observe that just upon keeping the V^V^ solutions in the media for 24 h at 37 °C, a decrease of 0.1–0.2 pH units occurs, while upon contact with the A2780 cells, the decrease in pH may be 0.3–0.4 pH units. Assuming that, concerning vanadium uptake, A2780 cells may behave similarly to erythrocytes, this could contribute to some increased vanadium uptake, but possibly cannot account for the whole difference observed in Figure 9.

**Table 2:** Measurement of pH Values of RPMI-1640 Incubation media with and without Addition of Solutions Containing V^V^ Anions, with and without Contact with A2780 Cells^a^

media and conditions	pH values measured^b^
50 μM ‘V_10_ solution’ without preincubation with the media	8.06 (I) - 8.07 (F)
50 μM ‘V_1_ solution’ without preincubation with the media	8.05 (I) - 8.06 (F)
50 μM ‘V_10_ solution’ after 24 h of incubation with the media at 37 °C	7.99 (I) - 7.97 (F)
50 μM ‘V_1_ solution’ after 24 h of incubation with the media at 37 °C	7.82 (I) - 7.90 (F)
supernatants after 24 h of incubation at 37 °C with A2780 cells	
control (only media, without addition of V^V^)	7.65 (I) - 7.65 (F)
50 μM ‘V_10_ solution’ without preincubation with the media	7.70 (I) - 7.71 (F)
50 μM ‘V_1_ solution’ without preincubation with the media	7.68 (I) - 7.69 (F)

a Note that the total vanadium concentration corresponding to all these experiments is approximately equal.

b Initial (I) and final (F) pH values measured are indicated after further 24h of standing.

The apparent higher V uptake in the experiments with previous incubation of the vanadate solutions with cells may be partly correlated with the cellular viability observed in these experiments, since there is loss of viability between the conditions without preincubation and with preincubation. In Figure S16, we depict photographs of A2780 cells upon their incubation in conditions equivalent to those corresponding to Figure 9. If there is a significant loss of the number of A2780 cells, as is the case comparing the red and blue columns, the number of living cells, those collected for vanadium uptake measurements, is much lower than in the case of the experiments with preincubation (red columns). Therefore, the vanadium present in the media will be more concentrated in the available living cells; thus, the total amount determined by ICP-MS will be higher, as was indeed observed. Probably, this effect is more relevant to explaining the different global vanadium uptake observed between the red and blue columns in Figure 9 than the pH effects discussed above.

### 3.8 ^51^V NMR Experiments with Vanadate­(V) Solutions

Several types of ^51^V NMR experiments were carried out with oxidovanadate­(V) solutions to evaluate the relative concentrations of V^V^-containing species present in the cell medium before and after contact with A2780 cells.

Figure 10(A) depicts the ^51^V NMR spectrum of a ‘V_10_ solution’ ([V]_total_ = 720 μM) in RPMI-1640 cell medium containing 2% FBS. After ∼3 h at 37 °C, the solution was placed in ice at ∼0 °C for about 3 h. Upon letting the solution warm up to room temperature, D_2_O was added so that its total content was 10% (v/v) (as mentioned in Section 2, this was done for all samples measured by ^51^V NMR), and the ^51^V NMR spectrum was recorded. The peaks at δ_V_ ≈ −513, ≈ −496 and ≈ −420 ppm, due to V_10_ anions, corresponding to V10A, V10B, and V10C, respectively, are clearly visible. The peak at ≈ −553 ppm is due to V_1_, its broadening possibly being partly due to the presence of some amount of V_2_. A similarly prepared solution was incubated with A2780 cells for 3 h at 37 °C. The supernatant of the medium was separated from the cells and kept on ice at ∼0 °C for about 3 h. The solution was inserted in a NMR tube, and the ^51^V NMR spectrum was recorded (Figure 10(B)). No peaks due to V_10_ anions are visible and the sharp peaks observed are due to V_1_ (−558 ppm), V_2_ (−571 ppm), and V_4_ (−577 ppm). Upon keeping the tube with this supernatant solution at room temperature for 18 h, its ^51^V NMR spectrum was measured again (Figure 10(C)). The two spectra of (B) and (C) are equal, and no peaks due to V_10_ anions are visible. In Figure S17, the spectra of Figures 10(B),(C) are depicted in a broader field range (ca. +2000 to −2000 ppm), confirming that no other bands are recorded and that no peaks due to V_10_ anions are visible. The nondetection of peaks due to V_10_ anions in the supernatant is a quite remarkable result. The [V]_total_ used was 720 μM, meaning that [V_10_] = 72.0 μM in the initial sample if all V would be in the form of decavanadates.

**Figure 10:**
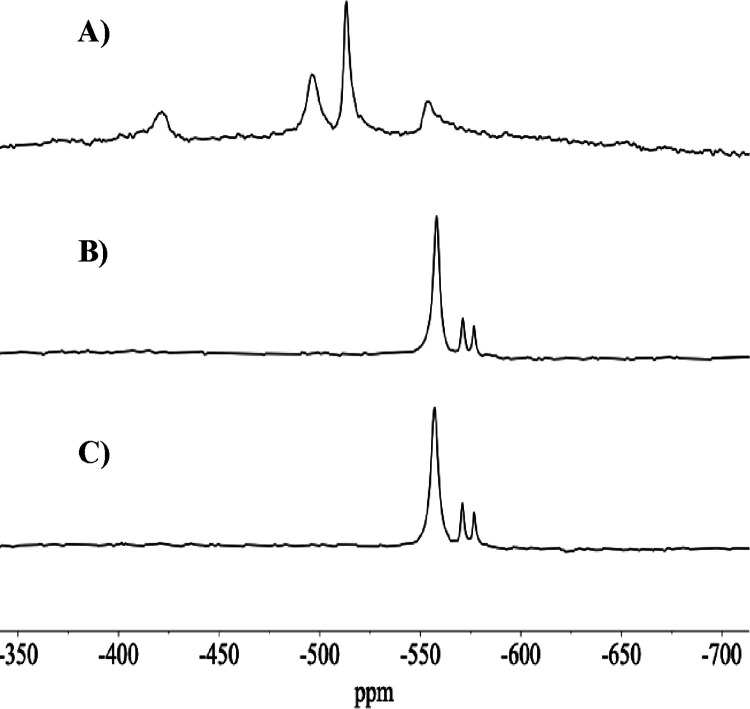
^51^V NMR spectra recorded with a sample consisting of a RPMI medium with 2% FBS to which a ‘V_10_ solution’ was added so that [V]_total_ = 720 μM. (A) After ∼3 h at 37 °C, the solution was placed in ice at ∼0 °C for about 3 h, and then the ^51^V NMR spectrum was recorded at room temperature (RT). The measured pH was 7.6. The peaks at δ_V_ ≈ −420, ≈ −496 and ≈ −513 ppm are clearly visible and are due to V_10_ anions (corresponding to V10C, V10B, and V10A, respectively). The peak at ≈ −553 ppm is due to V_1_, its broadening possibly being partly due to the presence of V_2_. (B) ^51^V NMR spectrum of the supernatant of an identical ‘V_10_ solution’ added to RPMI medium with 2% FBS ([V]_total_ = 720 μM) and separated from cells after incubation for 3 h with A2780 cells. No peaks due to V_10_ anions are visible and the sharp peaks observed are due to V_1_ (−558 ppm), V_2_ (−571 ppm), and V_4_ (−577 ppm). (C) Spectrum measured with the same solution of the spectrum of (B) upon keeping it at room temperature for an additional ∼18 h.

Similar experiments were carried out with a ‘V_10_ solution’ with [V]_total_ = 800 μM and the corresponding ^51^V NMR spectra are depicted in Figure S18. An adequate amount of the ‘V_10_ solution’ was added to RPMI-1640 media containing 2% FBS, so that [V]_total_ = 800 μM. After waiting for∼3 h at 37 °C, the solution was placed on ice at ∼0 °C for about 3 h. The sample was removed from ice and inserted in a NMR tube, and (A) the ^51^V NMR spectrum was recorded at RT. (B) After an additional 18 h at RT, the ^51^V NMR spectrum was recorded again. The peaks of decavanadates, visible at *t* = 0 h in Figure S18­(A), almost disappear at *t* = 18 h (only the peak due to V10A at −512 ppm is clearly visible). Therefore, in these experiments that were done using relatively high oxidovanadium­(V) concentrations (720 and 800 μM), it is clear that for solutions added to an RPMI-1640 media also containing 2% FBS, V_10_ anions may persist for several hours and may be detected by ^51^V NMR. However, if the same solution is placed in contact with the A2780 cells, after a relatively short time no decavanadate peaks are detected. For lower oxidovanadium­(V) concentrations, we predict that V_10_ anions decompose much faster.

Similar experiments were carried out either not using or using a higher % of FBS (10%) added to the RPMI-1640 medium. Figure 11 shows the ^51^V NMR spectra obtained. Figure 11(A) depicts the ^51^V NMR spectrum of a ‘V_10_ solution’, with [V]_total_ = 800 μM, upon its addition to a RPMI-1640 cell medium with no added FBS. Rather sharp and intense decavanadate and V_1_ peaks are visible (small amounts of V_2_ are also detected). Figure 11(B) depicts the spectrum of a similarly prepared sample but also containing 10% FBS; decavanadate peaks are visible but quite broad and weak, possibly due to binding of V_10_ anions to the BSA present in the cell medium. Figure 11(C),(D) depicts ^51^V NMR spectra of the supernatant of a ‘V_10_ solution’, with [V]_total_ = 800 μM, added to RPMI-1640 medium with 10% FBS, and upon incubation with A2780 cells, (C) upon 1 h of incubation with cells, and (D) the same but after upon 3 h incubation with the A2780 cells. The two spectra (C) and (D) are basically equal, and no peaks due to V_10_ anions are visible. Only rather sharp peaks due to V_1_ (strong), V_2_, and V_4_ are seen. The solution of the sample corresponding to (D) was kept for ∼18 h at RT and then the ^51^V NMR spectrum was measured. Again, only rather sharp peaks due to V_1_ (strong), V_2_, and V_4_ are seen and no peaks due to V_10_ anions are visible. These observations are similar to those reported in the hereby related experiments. The [V]_total_ used was 800 μM, meaning that [V_10_]_total_ = 80.0 μM in the samples at the start of the experiments, assuming that all V^V^ would be in the form of decavanadates.

**Figure 11:**
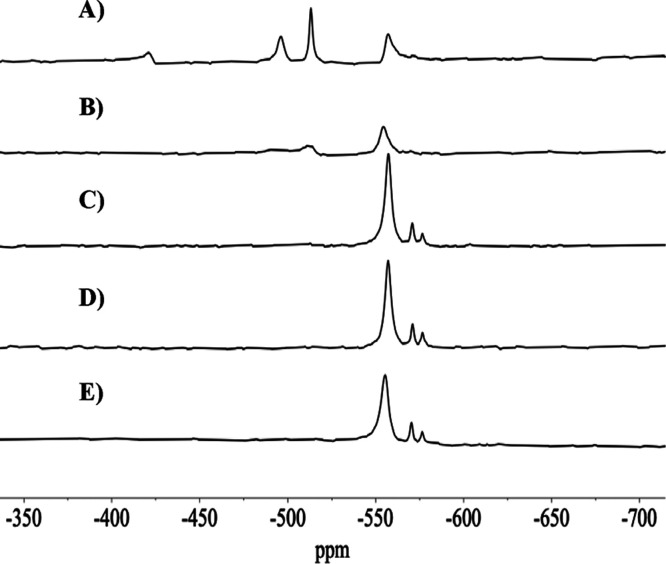
(A) ^51^V NMR spectrum of a ‘V_10_ solution’ ([V]_total_ = 800 μM) added to a RPMI cell medium with no FBS; (B) ^51^V NMR spectrum of a ‘V_10_ solution’ ([V]_total_ = 800 μM) ∼15 min after its addition to a RPMI cell medium with 10% FBS. (C) ^51^V NMR spectrum of the supernatant of the ‘V_10_ solution’ added to a RPMI cell medium with 10% FBS, separated upon incubation for 1 h with A2780 cells. (D) ^51^V NMR spectrum of the supernatant of ‘V_10_ solution’ added to an RPMI cell medium with 10% FBS and separated after incubation for 3 h with A2780 cells. (E) The same solution of (D) was retained at room temperature for further 18 h. In (C)–(E), only rather sharp peaks due to V_1_ (strong), V_2_, and V_4_ are seen, but no peaks due to V_10_ anions are visible.

It is clear that for ‘V_10_ solutions’, with [V]_total_ = 800 μM, added to an RPMI-1640 media also containing 10% FBS, V_10_ anions may persist for several hours. However, as in the similar experiments having 2% FBS, if the same solution is placed in contact with the A2780 cells, after a relatively short time no decavanadate peaks are detected. Whether the nondetected V_10_ anions bind to the cell membranes or not is not known, but comparing the intensities of all ^51^V NMR spectra, apparently the main process that takes place is a much faster hydrolysis of decavanadates in the presence of A2780 cells than in equivalent conditions in the absence of cells. Nevertheless, we cannot rule out the fast binding of small amounts of V_10_ anions to the cell membranes. For lower oxidovanadium­(V) concentrations, we predict that V_10_ anions decompose much faster. In all samples that were in contact with the A2780 cells, the ^51^V NMR spectra show no peaks due to V_10_ anions and only bands due to V_1_, V_2_, and V_4_ are detected.

Figure S19 depicts the ^51^V NMR spectrum of a ‘V_10_ solution’ with ([V]_total_ ≈ 800 μM added to RPMI-1640 media (with no FBS)). In Figure S19­(A), the spectrum of the sample corresponding to Figure 11(A) is included; as mentioned, rather sharp and intense peaks due to V_10_ and V_1_ anions are visible. Figure S19­(B) depicts the spectrum of the same solution after keeping it at RT for ∼18 h. The peaks due to V_10_ anions totally (or almost) disappeared; a very weak band at ∼ −512 ppm is apparently visible, which could be assigned to V10C, and sharp peaks due to V_1_ (strong), V_2_, and V_4_ are clearly recorded.

Globally, the ^51^V NMR data recorded with the ‘V_10_ solution’ added to the RPMI-1640 media, containing or not FBS, confirm that the V_10_ anions decompose faster in these media than in aqueous solutions at pH ∼ 7–8. Moreover, the data also indicate that no V_10_ anions are found in the supernatant of the RPMI-1640 cell medium that was placed in contact with cells. However, our experiments cannot either confirm or rule out if decavanadates might be bound to the cell membranes or were relevant for the vanadium uptake (or biological effect). It is possible that upon incubation of cells with a solution containing V_10_ anions, these may bind to the cell membranes, this being potentiated or not by certain cations. However, if this occurs with A2780 cells, the viability of cells was not significantly affected when comparing the outcome of experiments with incubation with equivalent solutions not containg decavanadates. Nevertheless, if indeed some amounts of decavanadate anions bind to cell membranes, this may induce cell signaling, namely, if redox reactions, demonstrated above to be thermodynamically viable, take place. Whether this may help explain some of the biological effects reported for decavanadate compounds cannot be assessed from our data, but it is a field that deserves further studies.

## 4 Conclusions

The protonation and redox behavior are important characteristics of decavanadates with regard to their properties. A lower or greater propensity for the binding of V_10_ clusters to cell membranes (or other targets), or to be involved in redox processes may be fundamental for the type and relevance of their biological effects. The isolation and characterization by SC-XRD of three decavanadate anions, two with 4-dimethylaminopyridinium and one with 1-methylimidazolium cations, the analysis of the Hirshfeld surfaces, and the associated 2D fingerprint plots, attest the propensity of V_10_ anions to establish hydrogen bonds (and van der Waals interactions), as is expected for V_10_ anions.^165^

DFT calculations further demonstrate the tendency for protonation of the decavanadates, and for the first time, the possibility of reduction (V^V^ → V^IV^) by one or two electrons occurring with the maintenance of the structure of the V_10_ cluster is also confirmed. The natural tendency of the V_10_ species to gain electrons increases if ion pairing occurs, which is notable if H.4-Me2AmPy^+^ is the counterion and modest with H.1-MeIm^+^. The mentioned ion pairing hampers protonation of the decavanadate. Ion-pairing trends are reproduced for V_2_ and V_4_ species, whereas V_1_ does not present ion pairing with either counterion analyzed. Considering the energies computed for proton-coupled electron transfer processes, they indicate the different stabilities for the V_10_ derivatives and the prevalence of the [HV^V^_9_V^IV^]^6–^, [H_2_V^V^_8_V^IV^_2_]^6–^, or [H_2_V^V^_9_V^IV^]^5–^ species over V_10_. These conclusions support the complexity of the factors to be analyzed when trying to understand any particular biological effect observed with solutions containing decavanadates: protonation, redox processes, hydrolysis, and role of counterions are all among the phenomena that must be taken into account.

It is shown that decavanadates at [V]_total_ in the range 720–800 μM (this corresponds to [V_10_] = 72–80 μM) undergo extensive hydrolysis when added to RPMI-1640 cell incubation media at pH 7–8 containing 2 or 10% FBS (Figures 10 and 11 and S17–S19); in fact, within relatively short periods of time (ca. 3–4 h) the ^51^V NMR peaks due to V_10_ anions disappear, and only peaks due to V_1_, V_2_, and V_4_ are clearly detected. If the RPMI-1640 cell media do not contain FBS, upon addition of a ‘V_10_ solution’ with ([V]_total_ ≈ 800 μM), initially rather sharp and intense decavanadate and V_1_ peaks are visible, but after ∼18 h the peaks due to V_10_ anions disappear. With more diluted V_10_ solutions, hydrolysis is certainly more extensive and occurs faster, but ^51^V NMR peaks may become too weak to draw reliable conclusions from the spectra measured.

Remarkably, when the ‘V_10_ solutions’ were added to the same RPMI-1640 cell media, either containing 2 or 10% FBS, upon 1 or 3 h incubation with A2780 cells, no peaks due to V_10_ anions were detected in the supernatant. Instead, quite sharp V_1_ (strong), V_2_, and V_4_ peaks are detected. The ^51^V NMR spectra of these samples do not change upon further keeping the solutions at room temperature for up to 18 h. These results place doubts on assigning which are the V-containing species responsible for the biological effects observed. In fact, these effects may be due to decavanadate, but also to any of the several other V-containing species present, namely, those that may form with components of the incubation medium.

Importantly, this conclusion is reinforced in experiments with samples with equal total vanadium concentrations. It was observed that the cytotoxic effects on A2780 cells do not significantly differ in the experiments where solutions containing or not containing V_10_ anions were tested. This was also observed in the experiments with and without previous incubation of the solutions during 24 h with the RPMI-1640 media before their contact with the A2780 cells. The cellular viability also is not correlated with the higher or lower V uptake.

Therefore, we conclude and emphasize that interpretation of any type of biological effects observed with solutions ‘containing decavanadates’ must take into account the speciation of vanadium in the specific conditions of the experiments, this including possible interactions with organic cations present and possible redox reactions involving the vanadium species present. We only carried out this type of assay with A2780 cells, but we anticipate that similar conclusions may be extrapolated to other types of mammalian cells.

Regarding the use of decavanadate salts of organic cations in cell viability experiments, we emphasize that it is important to also test the viability of the free organic compounds and evaluate if any synergistic effect may be observed upon the use of 'solutions of decavanadate' or solutions of V^V^ not containing decavanadates. In these cases, if a synergistic effect is observed, the interpretation of data should be done also considering possible interactions of the organic cations with each of the V-containing species present, not only V_10_ anions. Moreover, regarding the interpretation of the behavior of V_10_ compounds *in vivo*, unless these are incorporated in some form of nanotype carriers, the maintenance of their structure is doubtful and should also be properly evaluated.

The crucial point for future research work evaluating the effects of decavanadates is to always try to understand which are the species that may be the relevant ones for the biological effect observed and not to simply assume that the effects are due to the V_10_ anions. Another aspect is to make very clear which are the concentrations used; it should be specified if the concentrations correspond to those of POV anions, or to the total vanadium concentration. In this work, we normally specified the total vanadium concentration.

## Supplementary Material



## Notes

^51^V NMR spectra of decavanadate solutions in several distinct conditions (stock solutions, in RPMI media containing or not fetal bovine serum and after contact with cells); Tables with selected bond lengths and angles; Hydrogen bonds results of bond valence sum calculations for compounds **1–3**; Figures depicting the structure and hydrogen bond network of compounds **1–3**; FT-IR spectra of compounds **1–3**; 2D fingerprint plots for compounds **1–3**; Histogram of different percentages of real interaction bonds, contacts and proportion surface of different atoms of compound **1–3**; Species distribution diagrams of solutions of oxidovanadate­(V) in the pH range 2–6 and for constant pH (=7.2); Radial distribution functions (RDF, *g*_COM···COM_(*r*)) plotting the time average distribution of counterions in proximity of V_10_ unit; Table with the amount of ion pairing for the two counterions studied and the several vanadate species examined; Cell viability data; Photographs of A2780 cells nontreated and upon incubation with V^V^-containing solutions (PDF)
