# Supplementary material for: Bioactivity of Decavanadate Compounds: Can Their In Vitro and In Vivo Effects Be Assessed in a Simple Manner?
Source: Inorg Chem. 2025 Oct 16;64(42):21004–24. doi: 10.1021/acs.inorgchem.5c03076 (PMC13089102; doi:10.1021/acs.inorgchem.5c03076)
Supplement: Supplementary file 1 [file ic5c03076_si_001.pdf]

Supporting Information

Bioactivity of decavanadate compounds. Can their in vitro and in vivo effects be assessed in a simple manner?

João Costa Pessoa,[1]* Rima Zarroug,[1,2-3] Nádia Ribeiro,[1] Isabel Correia,[1] Leonor Corte-Real,[1] Brahim Ayed,[2] Clara S. B. Gomes,[4-6] Fernanda Marques,[7] Albert Masip-Sánchez,[8] Marina Hernández-Carrasco,[8] Xavier López[8]*

[1] *Centro de Química Estrutural, Institute of Molecular Sciences and Departamento de Engenharia Química, Instituto Superior Técnico, Universidade de Lisboa, Av. Rovisco Pais, 1049-001 Lisboa, Portugal*

[2] *University of Monastir, Laboratory of Physico-chemistry of Materials LR01ES19, Faculty of Sciences of Monastir, 5000 Monastir, Tunisia.*

[3] *Department of Chemistry, Faculty of Sciences, University of Gabes, 6072 Gabes, Tunisia.*

[4] *LAQV-REQUIMTE, Department of Chemistry, NOVA School of Science and Technology, NOVA University Lisbon, Campus de Caparica, 2829-516 Caparica, Portugal*

[5] *UCIBIO, Department of Chemistry, NOVA School of Science and Technology, NOVA University Lisbon, Campus de Caparica, 2829-516 Caparica, Portugal*

[6] *Associate Laboratory i4HB, NOVA School of Science and Technology, NOVA University Lisbon, Campus de Caparica, 2829-516 Caparica, Portugal*

[7] *Centro de Ciências e Tecnologias Nucleares and Departamento de Engenharia e Ciências Nucleares, Instituto Superior Técnico, Universidade de Lisboa, Estrada Nacional 10, 2695-066 Bobadela LRS, Portugal*

[8] *Universitat Rovira i Virgili, Departament de Química Física i Inorgànica, Marcel·lí Domingo 1, 43007 Tarragona, Spain.*

João Costa Pessoa. Email: joao.pessoa@ist.utl.pt

Xavier López: Email: javier.lopez@urv.cat

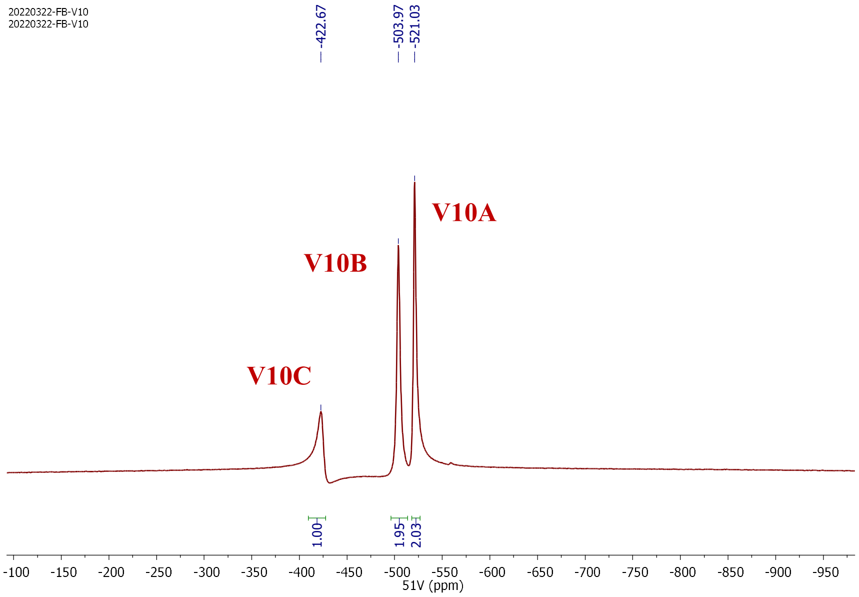

Figure S1. ^{51}V NMR spectrum of a V_{10} stock solution with $[V]_{total}$ = ~45 mM (90 % of the 'V_{10} solution' + 10 % D_2O) at pH = 4.0. Almost only decavanadate species are visible: the V atoms designated by V10C are at $\delta_V = -422.7$ ppm, those designated by V10B at −504.0 ppm and those designated by V10A at −521.0 ppm.

A similarly prepared solution, but after adding drops of a NaOH solution till pH = 5.3 already contains some amounts of V_1, V_2 and V_4 species (Figure S2).

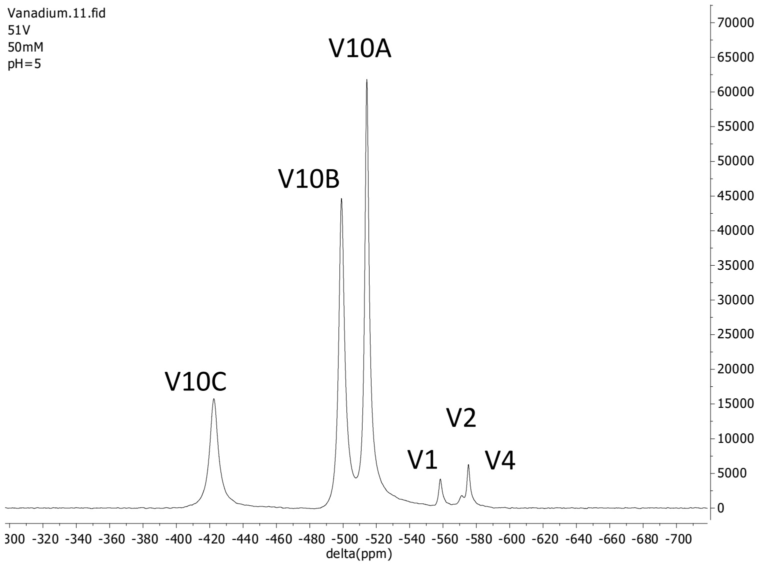

Figure S2. ^{51}V NMR spectrum of a V_{10} stock solution with [V]total = ~45 mM at pH = 5.3. Decavanadate species are the predominant ones, but V_1, V_2 and V_4 are also clearly visible.

Solutions used for experiments in the absence of decavanadates were prepared by dissolving $NaV^{V}O_3$ at relatively high pH, for example in NaOH ~0.001 or 0.01 M. These solutions were next diluted 1:10 or 1:100 with water, the pH becoming ca. 10.0. Figure S3 depicts the ^{51}V NMR spectrum of such a solution, used as stock solution for experiments in conditions decavanadates are not present in the media. Solutions prepared in this way were designated as 'V$_1$ solution' although, besides V_1, they may contain amounts of other V^{V}-species, but no decavanadate peaks are visible.

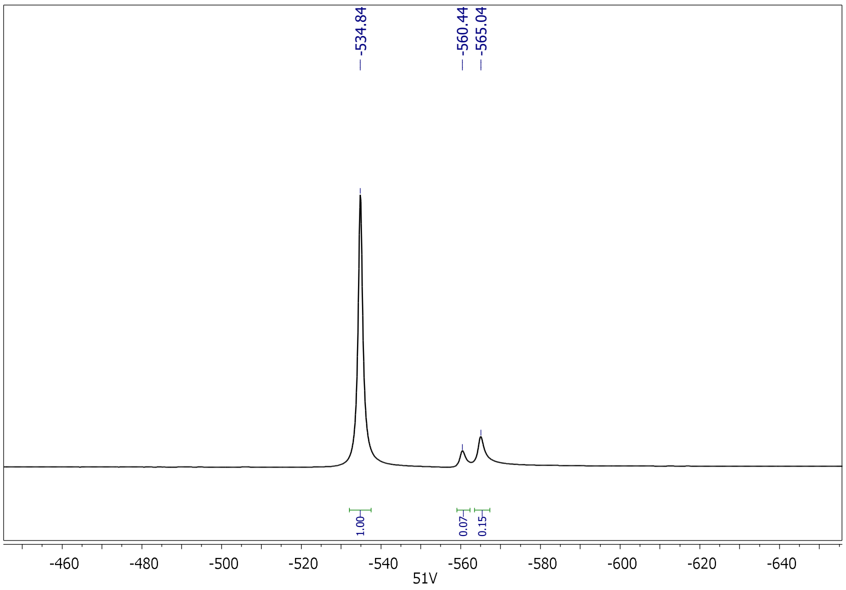

Figure S3. ^{51}V NMR spectrum of a 20.0 mM stock solution of $NaV^{V}O_3$ ('V$_1$ solution') at pH 10.0.

Table S1: Selected bond lengths [Å] and angles [°] compounds **1-3**.

**Compound (1)**			
Distances (Å)			
V1—O14	1.619 (2)	V3—O9	1.978 (2)
V1—O1	1.736 (2)	V3—O3	1.981 (2)
V1—O9	1.921 (2)	V3—O5	2.302 (2)
V1—O11^{i}	1.945 (2)	V4—O12	1.676 (2)
V1—O3	2.063 (2)	V4—O8^{i}	1.692 (2)
V1—O5^{i}	2.207 (2)	V4—O3^{i}	1.917 (2)
V2—O2	1.595 (2)	V4—O9	1.9543 (19)
V2—O6	1.829 (2)	V4—O5	2.0813 (19)
V2—O7	1.847 (2)	V4—O5^{i}	2.165 (2)
V2—O1^{i}	1.942 (2)	V5—O10	1.601 (2)
V2—O8	2.034 (2)	V5—O4	1.809 (2)
V2—O5	2.3273 (19)	V5—O6	1.818 (2)
V3—O13	1.599 (2)	V5—O11	1.962 (2)
V3—O7	1.832 (2)	V5—O12	2.059 (2)
V3—O4	1.837 (2)	V5—O5	2.300 (2)
Angles (º)			
O14—V1—O1	104.90 (10)	O13—V3—O5	175.94 (10)
O14—V1—O9	103.66 (10)	O7—V3—O5	80.13 (8)
O1—V1—O9	95.39 (9)	O4—V3—O5	79.65 (8)
O14—V1—O11^{i}	97.51 (10)	O9—V3—O5	75.58 (7)
O1—V1—O11^{i}	94.79 (10)	O4—V3—O3	154.56 (9)
O9—V1—O11^{i}	153.23 (9)	O9—V3—O3	77.19 (8)
O14—V1—O3	97.38 (9)	O12—V4—O9	95.62 (9)
O1—V1—O3	157.56 (9)	O8^{i}—V4—O9	96.50 (9)
O9—V1—O3	76.53 (8)	O3^{i}—V4—O9	155.71 (9)
O11i—V1—O3	84.74 (9)	O12—V4—O5	87.88 (9)
O14—V1—O5^{i}	170.71 (10)	O8^{i}—V4—O5	164.85 (10)
O1—V1—O5^{i}	83.30 (8)	O3^{i}—V4—O5	80.73 (8)

O9—V1—O5^{i}	79.53 (8)	O9—V4—O5	81.46 (8)
O11i—V1—O5^{i}	77.16 (8)	O12—V4—O5i	166.38 (9)
O3—V1—O5^{i}	74.71 (7)	O8^{i}—V4—O5^{i}	86.09 (9)
O2—V2—O6	104.73 (11)	O3^{i}—V4—O5^{i}	80.58 (8)
O2—V2—O7	101.70 (10)	O9—V4—O5^{i}	79.86 (8)
O6—V2—O7	91.80 (10)	O5—V4—O5^{i}	78.77 (8)
O2—V2—O1^{i}	102.48 (10)	O3—V3—O5	75.93 (8)
O6—V2—O1^{i}	90.73 (9)	O12—V4—O8^{i}	107.27 (10)
O7—V2—O1^{i}	154.18 (9)	O12—V4—O3i	100.07 (9)
O2—V2—O8	99.90 (11)	O8^{i}—V4—O3^{i}	96.39 (9)
O6—V2—O8	155.30 (9)	O10—V5—O4	104.75 (11)
O7—V2—O8	84.90 (9)	O10—V5—O6	104.80 (11)
O1^{i}—V2—O8	82.17 (9)	O4—V5—O6	93.42 (10)
O2—V2—O5	174.46 (10)	O10—V5—O11	99.39 (11)
O6—V2—O5	80.66 (8)	O4—V5—O11	153.94 (10)
O7—V2—O5	79.15 (8)	O6—V5—O11	89.76 (9)
O1i—V2—O5	75.92 (8)	O10—V5—O12	99.36 (11)
O8—V2—O5	74.68 (8)	O4—V5—O12	85.60 (9)
O13—V3—O7	101.83 (10)	O6—V5—O12	155.22 (9)
O13—V3—O4	103.69 (11)	O11—V5—O12	80.88 (9)
O7—V3—O4	93.19 (9)	O10—V5—O5	171.38 (10)
O13—V3—O9	101.95 (10)	O4—V5—O5	80.25 (8)
O7—V3—O9	154.50 (9)	O6—V5—O5	81.64 (8)
O4—V3—O9	90.01 (9)	O11—V5—O5	74.63 (8)
O13—V3—O3	100.45 (10)	O12—V5—O5	73.78 (8)
O7—V3—O3	89.57 (9)		
Symmetry code: (i) -*x*+1, -*y*+2, -*z*+1.			
**Compound 2**			
Distances(Å)			
O7—V2	1.801 (3)	O11—V6	1.922 (3)
O7—V7	1.841 (3)	O11—V8	2.003 (3)
O8—V1	1.708 (3)	O11—V3	2.003 (3)

O8—V7	1.990 (3)	O12—V8	1.833 (3)
O9—V7	1.798 (3)	O12—V9	1.845 (3)
O9—V3	1.837 (3)	O13—V5	1.768 (3)
O10—V4	2.083 (3)	O13—V9	1.903 (3)
O10—V6	2.165 (3)	O14—V4	1.680 (3)
O10—V1	2.204 (3)	O14—V10	2.040 (3)
O10—V2	2.287 (3)	O15—V8	1.804 (3)
O10—V3	2.336 (3)	O15—V10	1.844 (3)
O10—V7	2.339 (3)	O16—V5	1.811 (3)
O19—V9	2.0911 (9)	O16—V10	1.898 (3)
O20—V9	1.601 (3)	O17—V10	1.602 (3)
O21—V4	1.864 (3)	O18—V9	1.796 (3)
O21—V3	1.967 (3)	O18—V10	1.849 (3)
O21—V8	2.015 (3)	O19—V6	1.6785 (8)
O22—V8	1.589 (3)	O23—V5	2.0988 (9)
O23—V4	2.0529 (8)	O3—V4	2.114 (3)
O1—V1	1.9473 (8)	O3—V5	2.215 (3)
O1—V2	1.9728 (8)	O3—V8	2.288 (3)
O2—V3	1.604 (3)	O3—V6	2.084 (3)
O24—V5	1.608 (3)	O23—V1	2.1389 (8)
O25—V4	1.681 (3)	O28—V1	1.621 (3)
O25—V2	2.050 (3)	O26—V2	1.831 (3)
O26—V3	1.820 (3)	O27—V2	1.608 (3)
Angles (º)			
O12—V8—O21	154.50 (14)	O11—V8—O3	75.34 (12)
O11—V8—O21	75.55 (13)	O21—V8—O3	75.55 (11)
O22—V8—O3	173.60 (15)	O22—V8—O15	105.07 (16)
O15—V8—O3	80.22 (12)	O22—V8—O12	102.53 (16)
O12—V8—O3	80.36 (13)	O15—V8—O12	94.69 (15)
O20—V9—O18	107.11 (17)	O22—V8—O11	98.82 (15)
O20—V9—O12	102.96 (16)	O15—V8—O11	153.78 (14)
O28—V1—O8	105.79 (16)	O12—V8—O11	90.48 (13)

O28—V1—O4	102.35 (15)	O22—V8—O21	100.65 (16)
O8—V1—O4	98.08 (14)	O15—V8—O21	89.39 (14)
O28—V1—O1	98.23 (12)	O17—V10—O15	103.07 (15)
O8—V1—O1	94.95 (11)	O17—V10—O18	103.68 (16)
O27—V2—O25	99.30 (15)	O15—V10—O18	92.20 (14)
O7—V2—O25	155.33 (14)	O17—V10—O16	101.00 (15)
O26—V2—O25	85.63 (13)	O15—V10—O16	154.53 (14)
O1—V2—O25	82.63 (9)	O18—V10—O16	90.12 (14)
O27—V2—O10	171.46 (14)	O17—V10—O14	99.66 (15)
O7—V2—O10	81.78 (13)	O15—V10—O14	84.69 (13)
O26—V2—O10	80.78 (13)	O18—V9—O12	93.45 (14)
O1—V2—O10	75.14 (8)	O20—V9—O13	100.92 (16)
O25—V2—O10	73.81 (12)	O18—V9—O13	91.90 (14)
O4—V1—O1	151.55 (10)	O12—V9—O13	152.76 (15)
O28—V1—O23	96.15 (11)	O20—V9—O19	97.65 (13)
O8—V1—O23	157.99 (11)	O18—V9—O19	155.14 (12)
O4—V1—O23	75.06 (9)	O12—V9—O19	82.90 (10)
O1—V1—O23	83.44 (3)	O13—V9—O19	81.11 (10)
O28—V1—O10	169.48 (14)	O20—V9—O3	170.20 (15)
O8—V1—O10	84.31 (13)	O18—V9—O3	82.49 (13)
O4—V1—O10	78.66 (12)	O12—V9—O3	77.94 (13)
O1—V1—O10	77.60 (8)	O13—V9—O3	76.31 (12)
O23—V1—O10	73.88 (8)	O19—V9—O3	72.69 (8)
O27—V2—O7	104.66 (16)	O7—V2—O26	94.08 (14)
O27—V2—O26	104.04 (16)	O27—V2—O1	99.23 (13)
O18—V10—O14	156.55 (13)	O7—V2—O1	87.79 (11)
O16—V10—O14	83.18 (13)	O26—V2—O1	155.33 (11)
O17—V10—O3	173.10 (15)	O16—V10—O3	76.50 (12)
O15—V10—O3	78.64 (12)	O14—V10—O3	73.75 (11)
O18—V10—O3	82.84 (13)	O2—V3—O26	103.83 (16)
O9—V3—O11	88.46 (13)	O2—V3—O9	102.50 (16)
O21—V3—O11	76.60 (13)	O26—V3—O9	93.55 (15)
O2—V3—O10	175.46 (16)	O2—V3—O21	101.25 (15)

O26—V3—O10	79.61 (13)	O26—V3—O21	91.01 (14)
O9—V3—O10	80.08 (13)	O9—V3—O21	153.96 (14)
O21—V3—O10	75.56 (11)	O2—V3—O11	101.10 (15)
O11—V3—O10	75.13 (12)	O26—V3—O11	153.91 (14)
O14—V4—O25	106.92 (15)	O14—V4—O23	93.61 (11)
O14—V4—O21	99.40 (14)	O25—V4—O23	93.53 (11)
O25—V4—O21	101.01 (15)	O21—V4—O23	156.68 (10)
O23—V4—O3	78.36 (8)	O14—V4—O10	164.31 (14)
O10—V4—O3	78.48 (12)	O25—V4—O10	87.15 (14)
O21—V4—O10	84.27 (13)	O14—V4—O3	86.78 (14)
O23—V4—O10	78.29 (8)	O25—V4—O3	164.63 (14)
O4—V5—O23	73.90 (9)	O21—V4—O3	83.10 (13)
O24—V5—O3	170.01 (15)	O13—V5—O16	98.24 (15)
O13—V5—O3	83.16 (13)	O24—V5—O4	99.09 (15)
O16—V5—O3	80.93 (12)	O13—V5—O4	91.55 (14)
O4—V5—O3	74.97 (12)	O16—V5—O4	152.68 (13)
O23—V5—O3	75.17 (8)	O24—V5—O23	95.57 (12)
O24—V5—O16	102.76 (15)	O13—V5—O23	156.33 (11)
O16—V5—O23	87.76 (11)	O24—V5—O13	105.27 (16)
O19—V6—O5	107.88 (11)	O5—V6—O10	85.81 (14)
O19—V6—O11	99.48 (10)	O11—V6—O10	80.94 (12)
O5—V6—O11	95.99 (14)	O4—V6—O10	78.73 (12)
O19—V6—O4	96.79 (10)	O3—V6—O10	77.31 (11)
O5—V6—O4	97.04 (14)	O8—V7—O5	80.17 (13)
O11—V6—O4	154.90 (13)	O6—V7—O10	174.71 (15)
O19—V6—O3	88.97 (9)	O9—V7—O10	80.77 (13)
O5—V6—O3	163.11 (14)	O7—V7—O10	79.51 (12)
O11—V6—O3	82.02 (12)	O8—V7—O10	75.06 (11)
O4—V6—O3	79.30 (12)	O5—V7—O10	74.44 (12)
O19—V6—O10	166.11 (9)	O6—V7—O9	103.63 (16)
O7—V7—O8	87.73 (14)	O6—V7—O7	102.91 (16)
O6—V7—O5	102.60 (16)	O9—V7—O7	93.86 (15)
O9—V7—O5	87.63 (14)	O6—V7—O8	100.23 (16)
O7—V7—O5	153.32 (13)	O9—V7—O8	155.07 (14)

**Compound 3**			
Distances(Å)			
V1—O12	1.6826 (17)	V3—O7[i]	1.9929 (17)
V1—O5	1.6881 (18)	V3—O2	2.0399 (18)
V1—O2	1.9257 (17)	V3—O1	2.2403 (17)
V1—O7	1.9380 (16)	V4—O13	1.5967 (19)

V1—O1^{i}	2.0881 (16)	V4—O11^{i}	1.8310 (17)
V1—O1	2.1112 (17)	V4—O3	1.8634 (18)
V2—O14	1.6054 (18)	V4—O8	1.8894 (17)
V2—O3^{i}	1.8140 (18)	V4—O5	2.0650 (17)
V2—O4	1.8209 (17)	V4—O1^{i}	2.3394 (17)0
V2—O2^{i}	1.9896 (17)	V5—O1	2.3083 (16)
V2—O7	2.0285 (17)	V5—O10	1.6101 (17)
V2—O1	2.2581 (16)	V5—O11	1.8339 (18)
V3—O9	1.6171 (17)	V5—O4	1.8340 (18)
V3—O8^{i}	1.7862 (17)	V5—O6	1.8879 (17)
V3—O6	1.8191 (17)	V5—O12	2.0461 (18)
Angles(º)			
O12—V1—O5	105.61 (8)	O8^{i}—V3—O2	154.99 (8)
O12—V1—O2	97.31 (8)	O6—V3—O2	88.04 (7)
O5—V1—O2	97.43 (8)	O7^{i}—V3—O2	75.54 (7)
O12—V1—O7	96.44 (8)	O9—V3—O1	173.47 (8)
O5—V1—O7	97.45 (8)	O8^{i}—V3—O1	81.40 (7)
O2—V1—O7	156.20 (7)	O6—V3—O1	79.69 (7)
O12—V1—O1^{i}	165.69 (8)	O7^{i}—V3—O1	76.25 (6)
O5—V1—O1^{i}	88.70 (7)	O2—V3—O1	74.90 (6)
O2—V1—O1^{i}	80.75 (7)	O13—V4—O11^{i}	104.34 (9)
O7—V1—O1^{i}	81.13 (7)	O13—V4—O3	102.25 (9)
O12—V1—O1	87.83 (7)	O11^{i}—V4—O3	92.68 (8)
O5—V1—O1	166.56 (7)	O13—V4—O8	102.18 (9)
O2—V1—O1	80.33 (7)	O11^{i}—V4—O8	90.43 (7)
O7—V1—O1	80.88 (7)	O3—V4—O8	153.76 (8)
O1^{i}—V1—O1	77.86 (7)	O13—V4—O5	100.24 (8)
O14—V2—O3^{i}	103.65 (9)	O11^{i}—V4—O5	155.38 (8)
O14—V2—O4	104.23 (8)	O3—V4—O5	83.48 (7)
O3^{i}—V2—O4	95.81 (8)	O8—V4—O5	82.92 (7)
O14—V2—O2^{i}	99.49 (8)	O13—V4—O1^{i}	174.06 (8)
O3^{i}—V2—O2^{i}	90.35 (7)	O11^{i}—V4—O1^{i}	81.55 (7)
O4—V2—O2^{i}	153.27 (7)	O3—V4—O1^{i}	77.98 (7)
O14—V2—O7	98.98 (8)	O8—V4—O1^{i}	76.72 (7)
O3^{i}—V2—O7	155.19 (7)	O5—V4—O1^{i}	73.86 (6)
O3^{i}—V2—O7	155.19 (7)	O10—V5—O11	105.84 (9)
O4—V2—O7	88.31 (7)	O10—V5—O4	102.98 (9)
O2^{i}—V2—O7	75.87 (7)	O11—V5—O4	92.70 (8)
O14—V2—O1	173.07 (8)	O10—V5—O6	100.94 (9)
O3^{i}—V2—O1	81.15 (7)	O11—V5—O6	89.83 (8)
O4—V2—O1	79.99 (7)	O4—V5—O6	154.25 (8)
O2^{i}—V2—O1	75.29 (6)	O10—V5—O12	97.04 (8)
O7—V2—O1	75.48 (6)	O11—V5—O12	156.98 (7)

O9—V3—O8[i]	103.99 (9)	O4—V5—O12	84.48 (7)
O9—V3—O6	103.18 (8)	O6—V5—O12	83.27 (7)
O8[i]—V3—O6	95.58 (8)	O10—V5—O1	171.52 (8)
O9—V3—O7[i]	99.73 (8)	O11—V5—O1	82.36 (7)
O8[i]—V3—O7[i]	91.30 (7)	O4—V5—O1	78.38 (7)
O6—V3—O7[i]	153.66 (8)	O6—V5—O1	76.57 (7)
O9—V3—O2	99.21 (8)	O12—V5—O1	74.68 (6)
Symmetry code: (i) -*x*+1, -*y*+1, -*z*+1.			

Table S2: Hydrogen bonds for compounds **1-3**.

**Compound 1**					
D—H···A	***D—H***	**H···A**	***D···A***	**<DHA**	***Symmetry of A***
N2—H2A···O3	0.821	1.887	2.691	166.25	
N4—H4A···O7	*0.808*	1.931	2.736	174.23	
O11—H11A···O14	*0.766*	1.960	2.708	176.79	[2+x, y. z]
**Compound 2**					
D—H···A	***D—H***	**H···A**	***D···A***	**<DHA**	***Symmetry of A***
N3—H3A···O11	0.869	1.957	2.826	179.91	
N4—H4A···O12	0.938	1.760	2.665	161.23	
N6—H6A···O13	0.880	2.072	2.831	143.95	
N8—H8A···O16	0.826	1.945	2.766	172.26	
O1—H1A···O24	0.821	1.899	2.696	163.38	[1-x, 1-y, 1-z]
O23—H23a···O28	0.955	1.753	2.680	162,57	[1-x, 1-y, 1-z].
**Compound 3**					
D—H···A	***D*—H**	**H···*A***	***D···A***	**<DHA**	***Symmetry of A***
N6-H6A···O7	0.965	1.716	2.660	165.16	[-x+1, -y+1, -z+1]
N2-H2A ··· O6	0.816	1.778	2.592	175.38	
N3-H3A ··· O2	0.882	1.758	2.622	165.70	[-x+1, -y+1, -z+1]
O17-H17B ··· O18	1.010	1.776	2.771	167.65	
O16-H16B ··· O17	0.820	2.059	2.866	168.40	
O15-H15B ··· O11	0.770	2.189	2.934	162.86	[x+1, y, z]
O16-H16A ··· O3	0.791	2.101	2.883	170.27	[-x+1, -y+1, -z+1]

O18-H18A··· O15	0.910	1.862	2.746	163.16	
O15-H15A ··· O9	0.702	2.192	2.878	165.59	[-x+1, -y+1, -z+1]
O18-H18B ··· O5	0.994	2.087	3.016	154.71	[x+1/2, -y+1/2, z+1/2]
O17-H17A ··· O10	0.903	2.125	3.017	169.29	[x+1/2, -y+1/2, z+1/2]
O17-H17A··· O12	0.903	2.556	3.082	117.78	[x+1/2, -y+1/2, z+1/2]

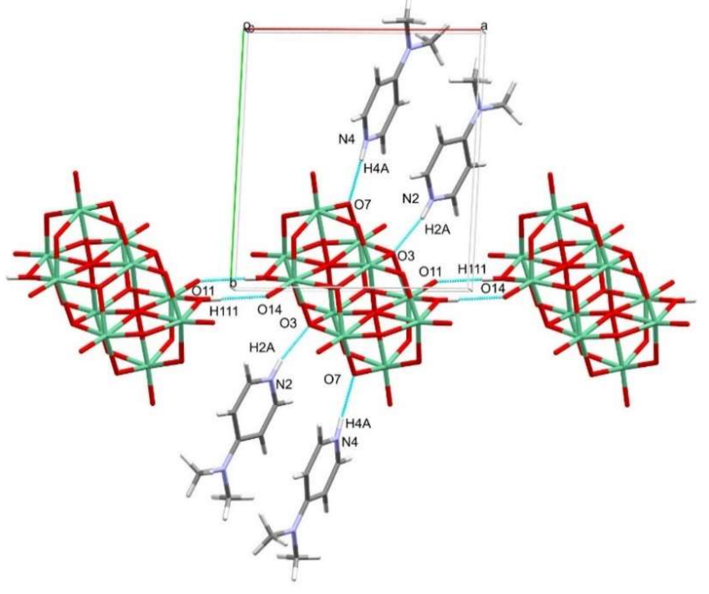

a
b
N4
H4A
O7
N2
H2A
O3
O11
H111
O14
O3
H2A
N2
O7
H4A
N4
O11
H111
O14

(A)

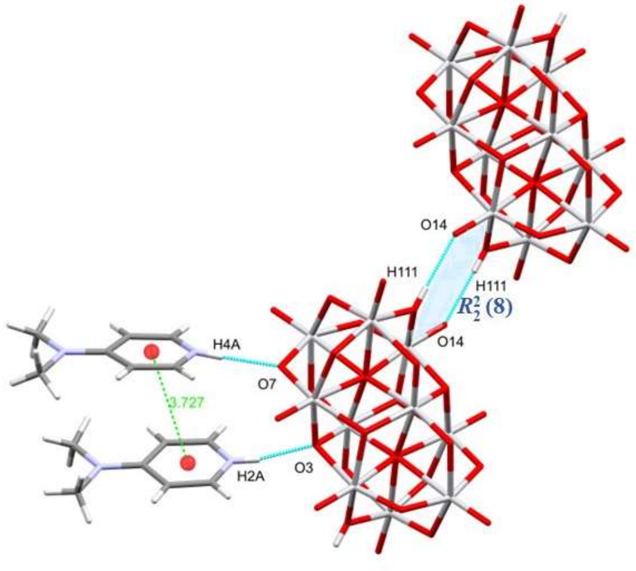

O14
H111
H111
R_2^2 (8)
O14
H4A
O7
3.727
H2A
O3

(B)

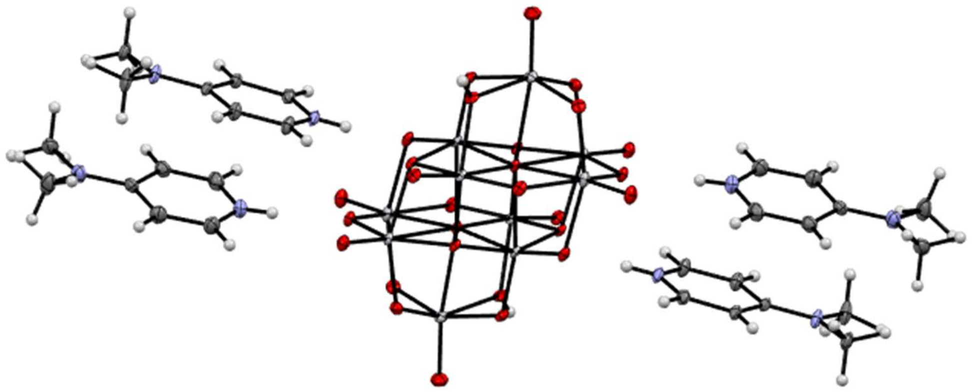

(C)

Figure S4. (A) Structure of (H.4-Me2AmPy)$_4$[$H_2V_{10}O_{28}$] (**1**), viewed along the *c* axis. The hydrogen bonds between H.4-Me2AmPy cations and the diprotonated decavanadate anion are shown in blue dashed lines. **(B)** Parallel offset π–π stacking between displaced H.4-Me2AmPy rings (green dashed lines) and a $R^2_2(8)$ ring motif highlighted. **(C)** ORTEP drawing of the structure of **1**, using 50 % level ellipsoids.

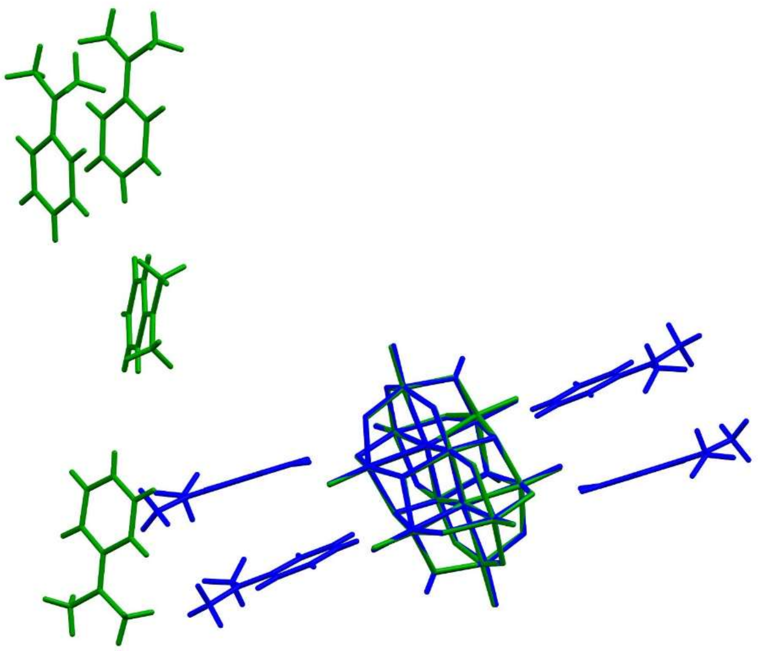

Figure S5. Superposition of the crystal structures of polymorphs **1** (blue) and **2** (green), drawn using Mercury[1] software.

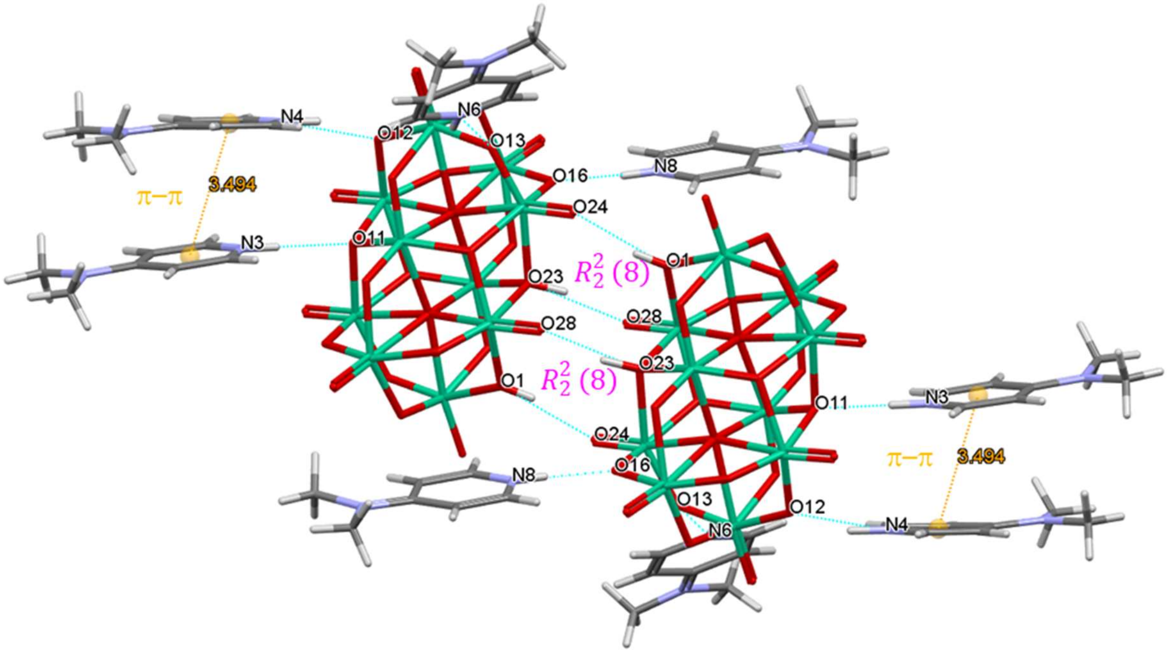
N4
O12
N6
O13
O16
N8
π−π
3.494
N3
O11
O24
O1
R_2^2 (8)
O23
O28
O28
O23
O1
R_2^2 (8)
O11
N3
O24
O16
N8
π−π
3.494
O13
O12
N6
N4

(A)

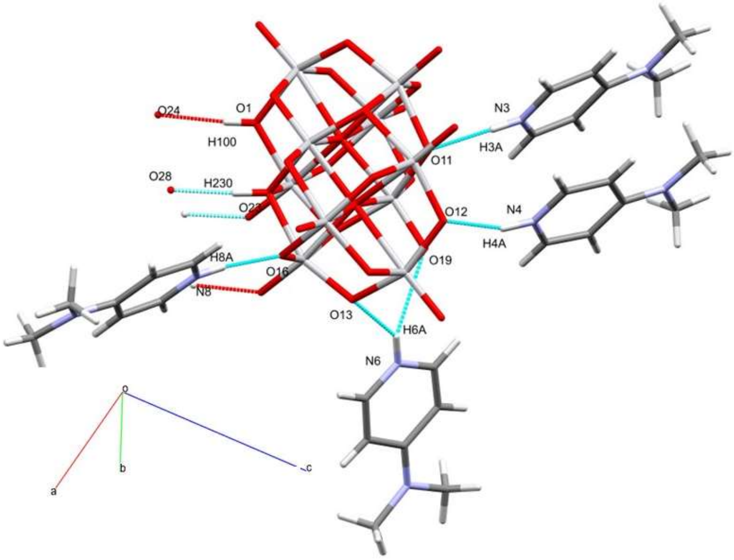
O24
O1
H100
N3
H3A
O11
O28
H230
O12
N4
H4A
H8A
O16
N8
O19
O13
H6A
N6
a
b
c

(B)

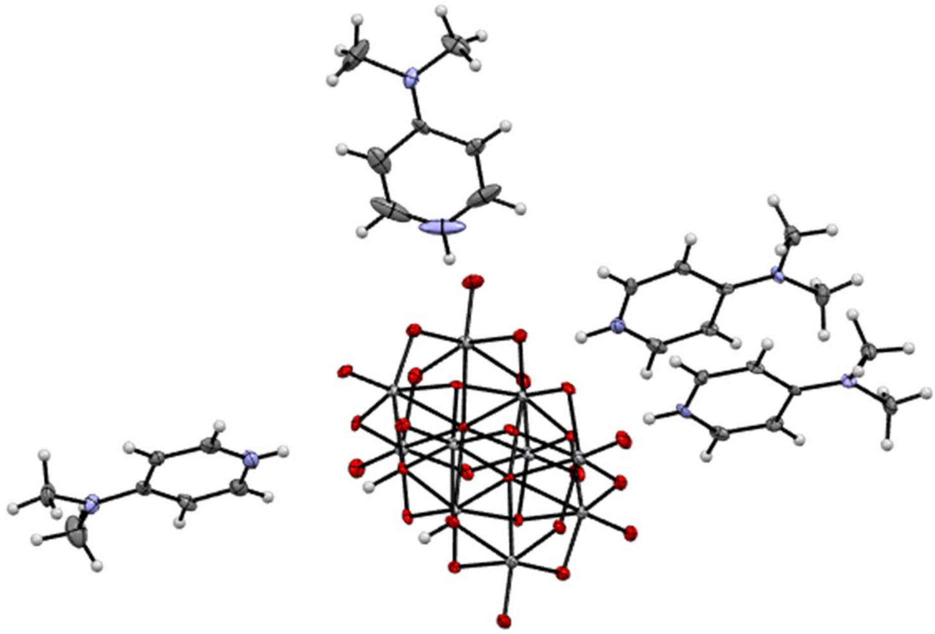

(C)

Figure S6. Several views of the 3D-Structure of $(H.4Me2AmPy)_4[H_2V_{10}O_{28}]$ (**2**). **(A)** A view of the network of hydrogen bonds of the compound; **(B)** Different view of the hydrogen bonds between H.4Me2AmPy cations and the diprotonated decavanadate anion shown in blue dashed lines; **(C)** Ortep drawing of the molecular structure of **2**, using 50 % level ellipsoids.

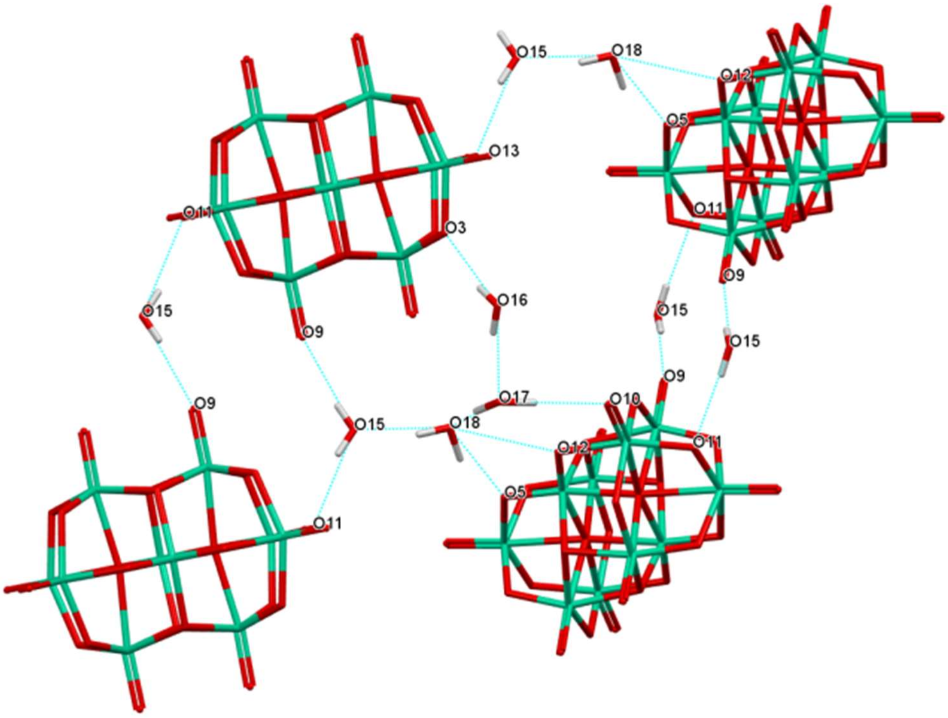
O15
O18
O12
O5
O13
O11
O3
O16
O9
O17
O10
O12

(A)

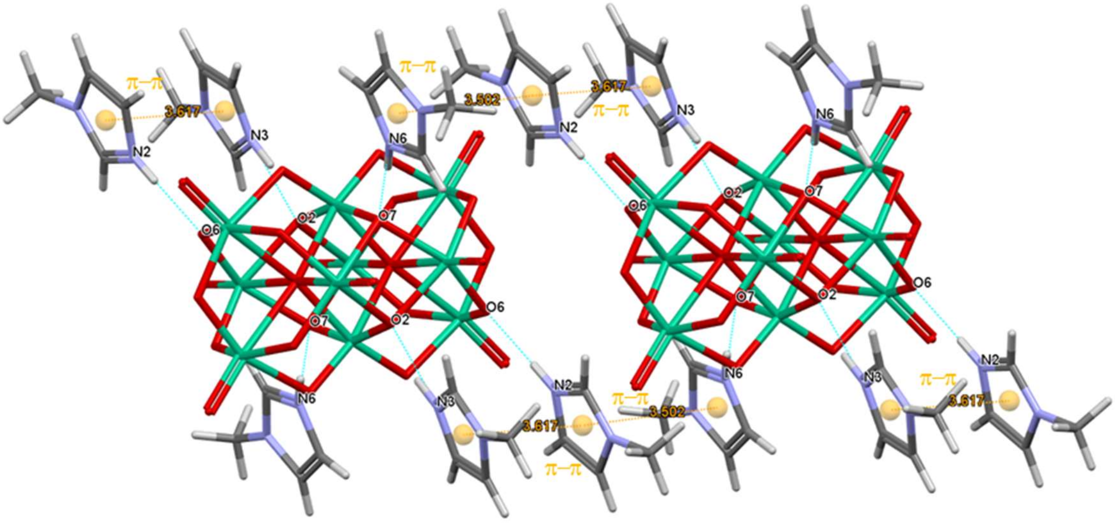
π–π
3.617
3.502
N2
N3
N6
O6
O2
O7

(B)

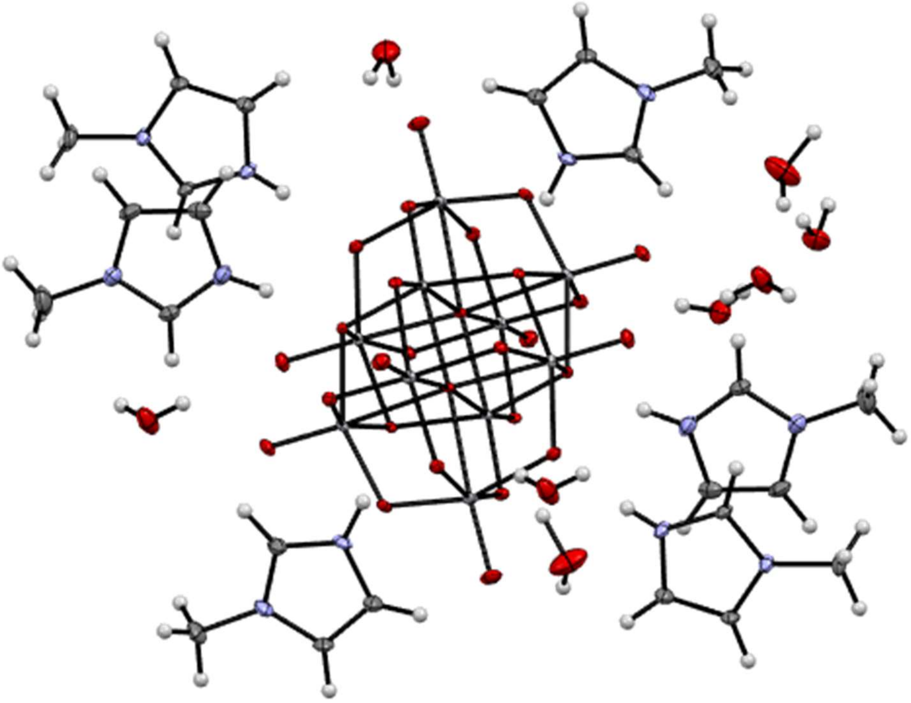

(C)

Figure S7. Single crystal X-ray diffraction structure of $(C_4H_7N_2)_6[V_{10}O_{28}]\cdot 8H_2O$ (**3**). **(A)** Network of hydrogen bonds O-H···O; **(B)** N-H···O and parallel offset π–π stacking between displaced *N*-methylimidazole rings; **(C)** Ortep drawing of the molecular structure of **3**, using 50 % level ellipsoids.

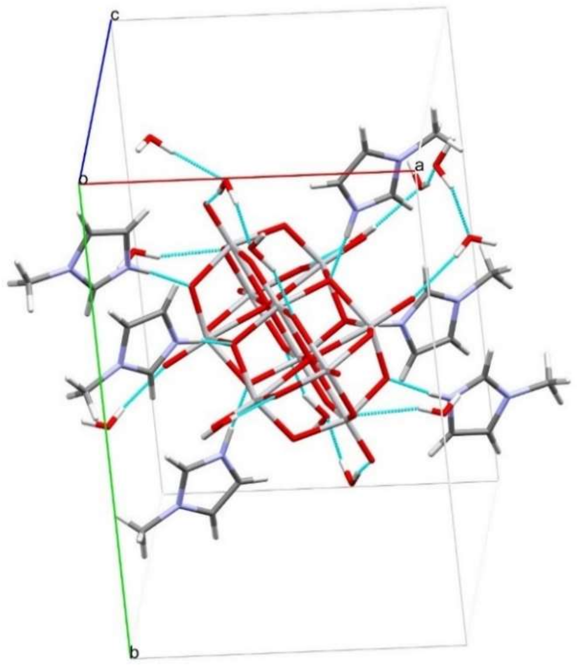

Figure S8. Supramolecular network of compound **3**.

Table S3. Results of bond valence sum calculations (A) for compound **1**;[2–4] (B) for compound **2**; (C) for compound **3**.

(A)							
**Atom**	**V1**	**V2**	**V3**	**V4**	**V5**	**Σ S_i(cal)**	**Σ S_i(th)**
**O1**	1.198	0.687				1.885	+2
**O2**		1.755				1.755	+2
**O3**	0.495		0.619	0.734		1.848	+2
**O4**			0.913		0.985	1.898	+2
**O5**	0.336	0.243	0.260	0.471	0.261	1.571	+2
**O6**		0.933			0.959	1.892	+2
**O7**		0.887	0.924			1.811	+2
**O8**		0.536		1.351		1.887	+2
**O9**	0.728		0.624	0.664		2.016	+2
**O10**					1.727	1.727	+2
**O11**	0.680				0.651	**1.331**	+2
**O12**				1.408	0.500	1.908	+2
**O13**			1.735			1.735	+2
**O14**	1.646					1.646	+2
**Σ S_i(cal)**	5.084	5.040	5.074	5.004	5.083		
**Σ S_i(th)**	+5	+5	+5	+5	+5		

(B)											
Atom	V1	V2	V3	V4	V5	V6	V7	V8	V9	V10	Σ S_i(cal)	Σ S_i(th)
O1	0.677	0.632									**1.309**	2
O2			1.715								1.715	2
O3				0.432	0.328	0.468		0.27	0.217	0.249	1.964	2
O4	0.764				0.576	0.677					2.017	2
O5						1.367	0.565				1.932	2
O6							1.771				1.771	2
O7		1.006					0.902				1.908	2
O8	1.29						0.604				1.894	2
O9			0.914				1.014				1.928	2
O10	0.338	0.271	0.237	0.47		0.376	0.235				1.927	2
O11			0.582			0.724		0.584			1.89	2
O12								0.923	0.891		1.814	2
O13					1.099				0.763		1.862	2
O14				1.396						0.527	1.923	2
O15								0.997		0.896	1.893	2

O16					0.98					0.773	1.753	2
O17										1.723	1.723	2
O18									1.021	0.882	1.903	2
O19						1.4			0.459		1.859	2
O20									1.726		1.726	2
O21			0.641	0.847				0.563			2.051	2
O22								1.783			1.783	2
O23	0.403			0.509	0.45						**1.362**	2
O24					1.692						1.692	2
O25		0.514		1.39							1.904	2
O26		0.929	0.954								1.883	2
O27		1.694									1.694	2
O28	1.636										1.636	2
Σ S_i(cal)	5.108	5.046	5.043	5.044	5.125	5.012	5.091	5.12	5.077	5.05		
Σ S_i(th)	5	5	5	5	5	5	5	5	5	5		

**(C)**							
Atom	V1	V2	V3	V4	V5	Σ S_i (cal)	Σ S_i (th)
O1	0.898	0.292	0.243	0.235	0.255	1.923	2
O2	0.718	0.604	0.455			1.777	2
O3		0.971		0.849		1.820	2
O4		0.953			0.92	1.873	2
O5	1.364			0.492		1.856	2
O6			0.907		0.795	1.702	2
O7	0.694	0.544	0.527			1.765	2
O8			1.006	0.792		1.798	2
O9			1.706			1.706	2
O10					1.684	1.684	2
O11				0.928	0.92	1.848	2
O12	1.385				0.518	1.903	2
O13				1.748		1.748	2
O14		1.706				1.706	2
Σ S_i (cal)	5.059	5.07	4.844	5.002	5.092		
Σ S_i (th)	5	5	5	5	5		

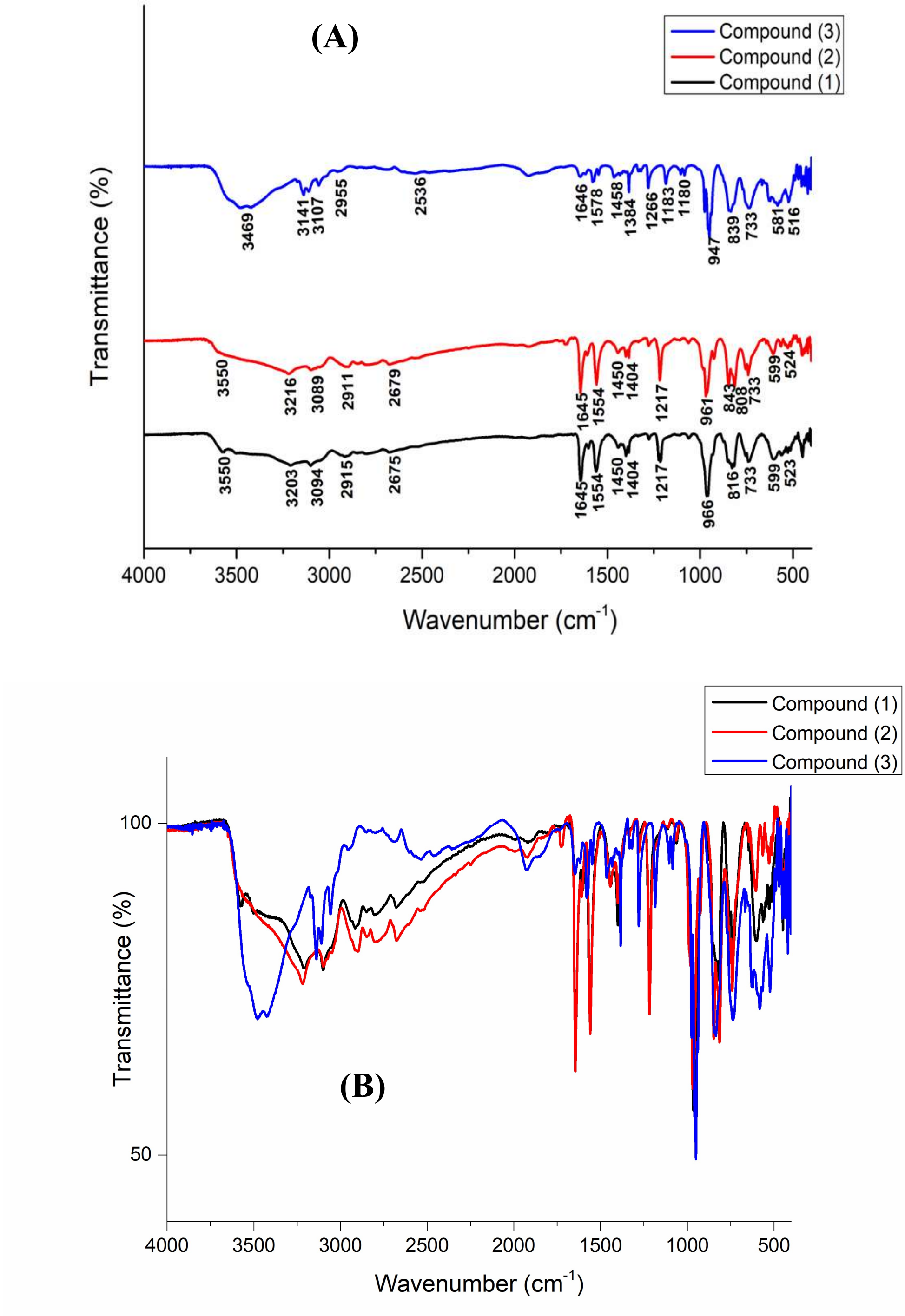

Figure S9 (A and B). FT-IR spectra of compounds **1-3**.

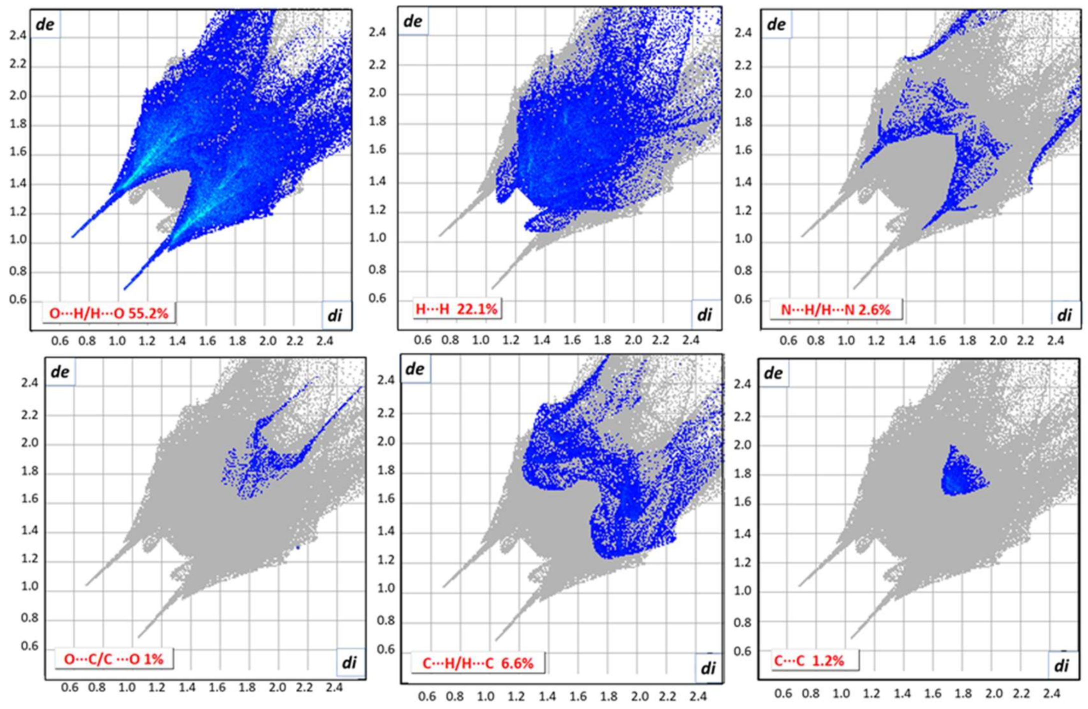
O···H/H···O 55.2%
H···H 22.1%
N···H/H···N 2.6%
O···C/C···O 1%
C···H/H···C 6.6%
C···C 1.2%

(A)

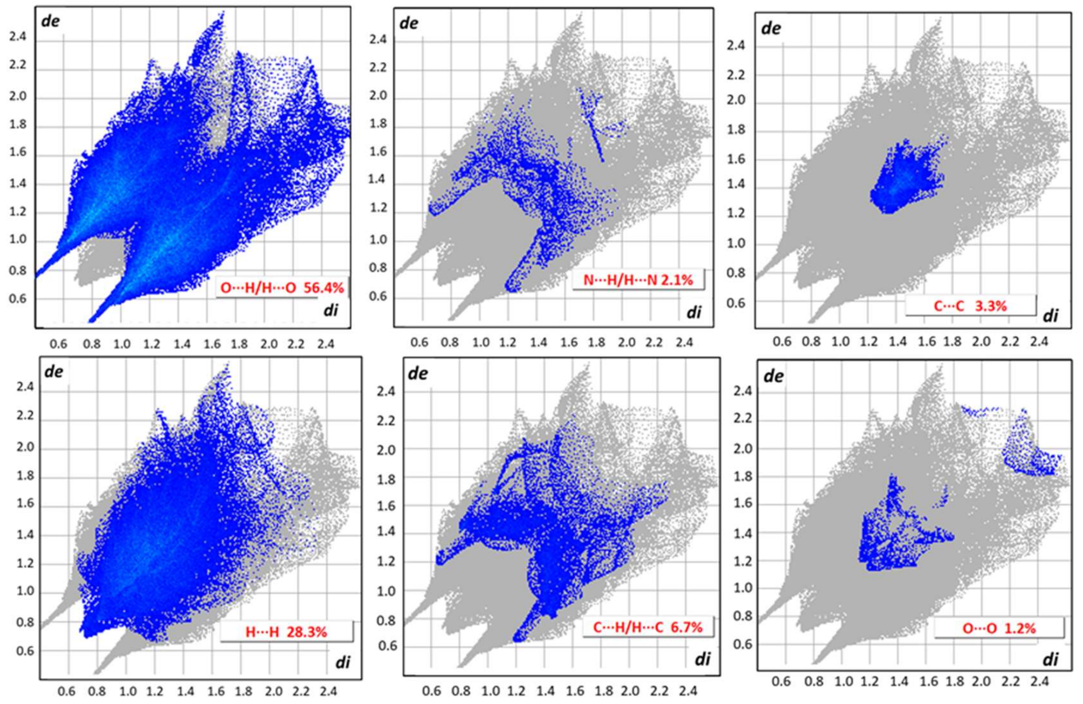
O···H/H···O 56.4%
N···H/H···N 2.1%
C···C 3.3%
H···H 28.3%
C···H/H···C 6.7%
O···O 1.2%

(B)

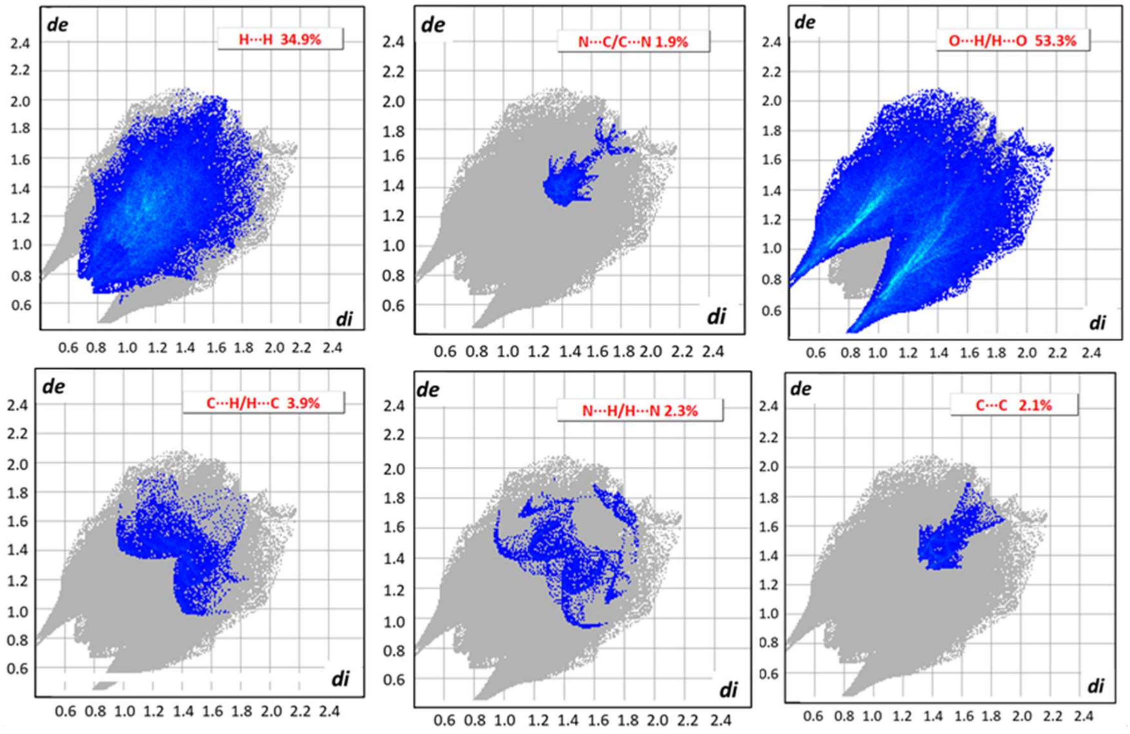

(C)

Figure S10. Important 2D finger print plots **(A)** of compound **1**; **(B)** of compound **2**; **(C)** of compound **3**.

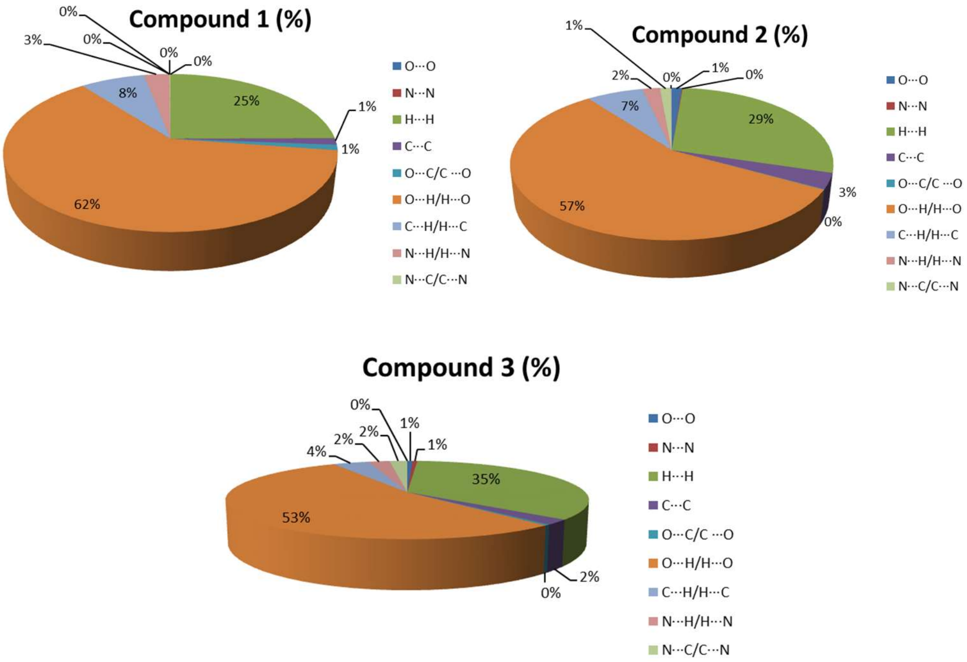

Figure S11. Histogram of different percentages of real interaction bonds, contacts and proportion surface of different atoms of compound **1-3**.

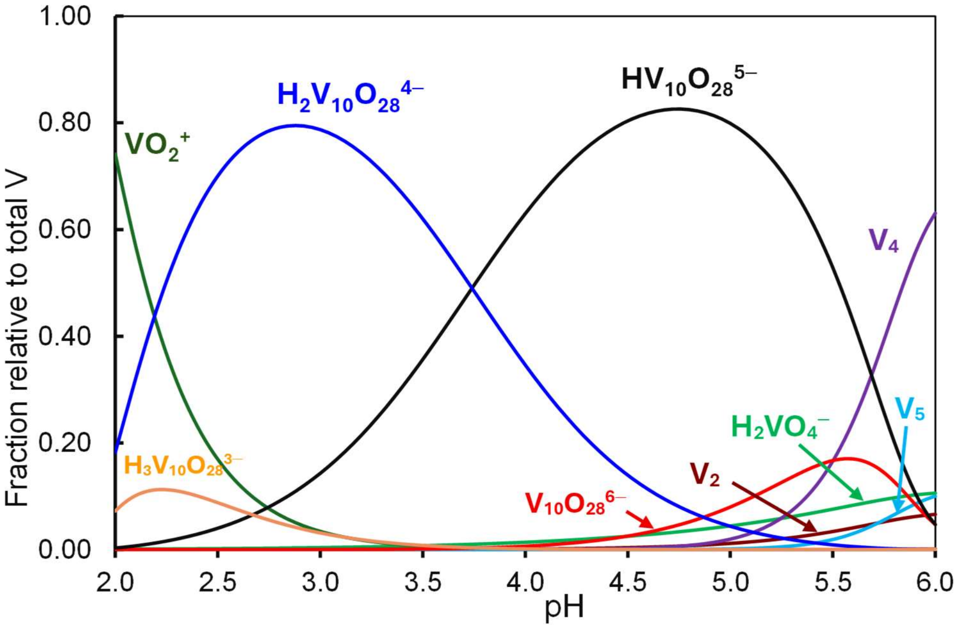

Figure S12. Species distribution diagram of solutions of oxidovanadate(V) in the pH range 2 - 6 for $[V]_{total}$ = 5.0 mM.

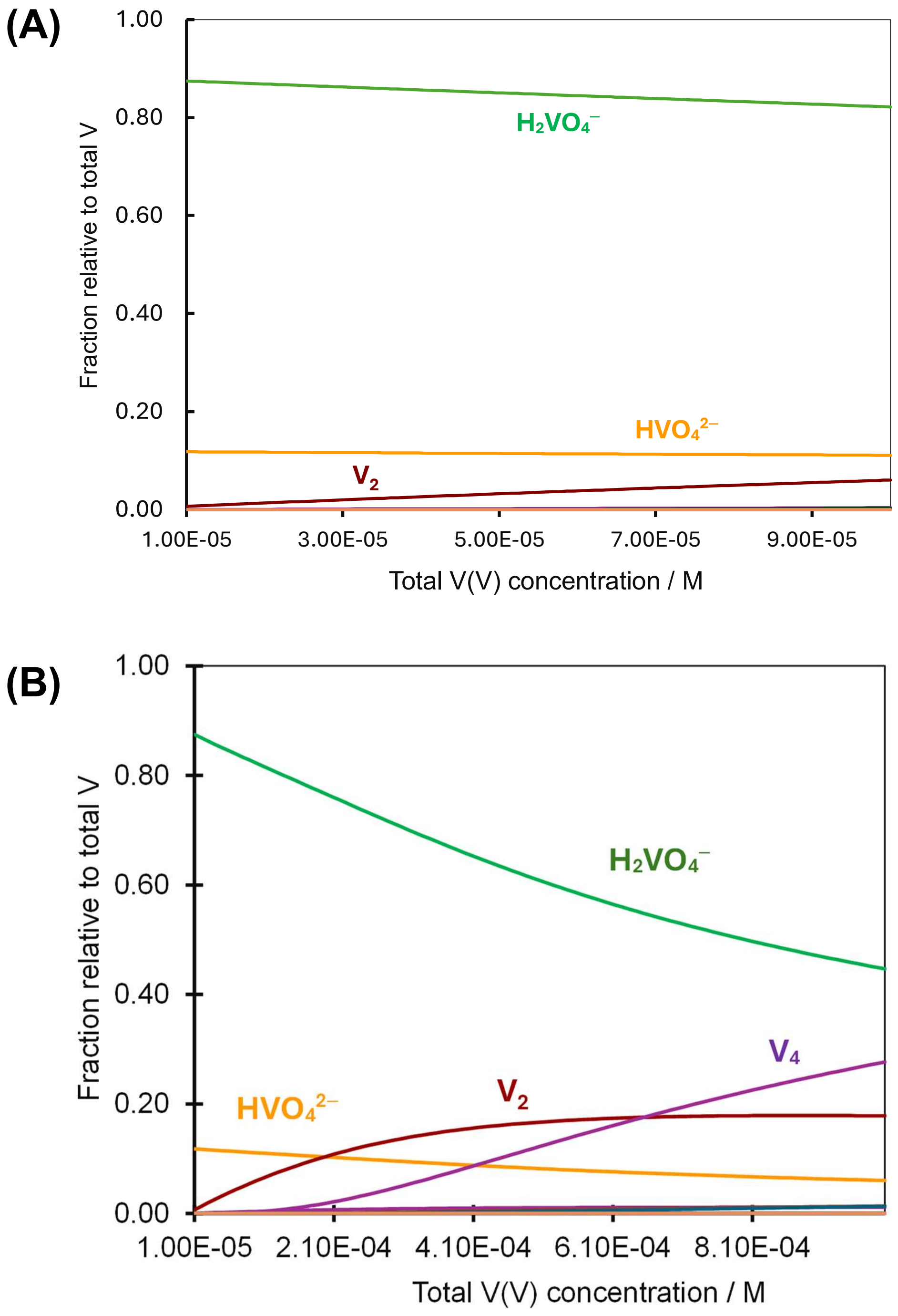

Figure S13. Species distribution diagram of solutions of oxidovanadate(V) at pH = 7.2 in the range of $[V]_{total}$ **(A)** from 10 to 100 μM; **(B)** from 10 to 1000 μM.

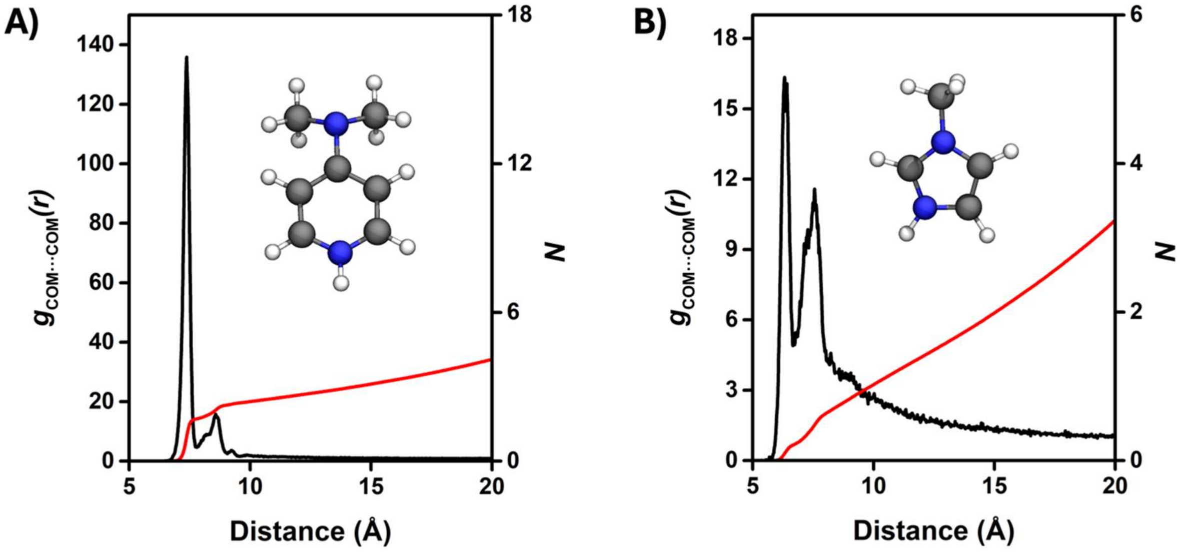

Cation	Peak position (Å)	*I* (Å)	*N*
H.4-Me_2AmPy	1st: 7.38	9.04	2.26

Cation	Peak position (Å)	*I* (Å)	*N*
H.1-MeIm	1st: 0.634<br>2nd: 0.758	9.54	0.94

Figure S14. Molecular dynamics simulations on the V_{10} species. The radial distribution functions (RDF, $g_{COM\cdots COM}(r)$) plot the time average distribution of counterions in proximity of V_{10} unit as the distance between their centers of mass (COM). A) H.4-Me2AmPy and B) H.1-MeIm counterions in aqueous solution. The integration values *N* capture the main differences between both cations.

Table S4. Molecular dynamics simulations on V_x species. The amount of ion pairing (*N*) is shown for the two counterions studied and the different vanadate species examined.

**Vanadate species**	**Cation**	***N***
V_1	H.4-Me2AmPy	0.17
	H.1-MeIm	0.14
V_2	H.4-Me2AmPy	2.62
	H.1-MeIm	0.30
V_4	H.4-Me2AmPy	2.52
	H.1-MeIm	0.70
V_{10}	H.4-Me2AmPy	2.26
	H.1-MeIm	0.94

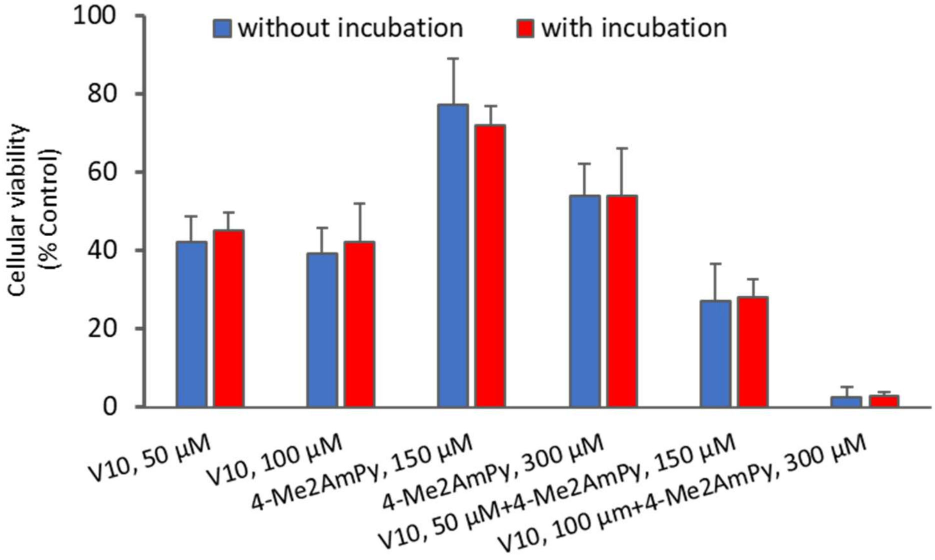

Figure S15. Cellular viability as % control (untreated cells) of V_{10} and 4-Me2AmPy, with or without previous incubation in cell medium for 24 h, at 37ºC before addition to the A2780 cells. Results are mean ± SD of two independent experiments done with four replicates. In these experiments it is clear that the cellular viability is not significantly different for fresh and aged solutions of these compounds. addition to the A2780 cells. Results are mean ± SD of two independent experiments done with four replicates. Note that the total vanadium concentration for each set of columns is approximately equal. For example, for the set of columns specifying 200 µM, for this means that the amount of decavanadate initially added corresponds to a concentration of 20 µM.

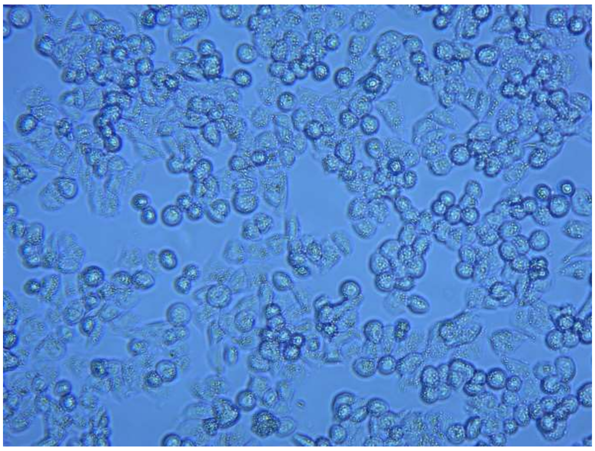

(A)

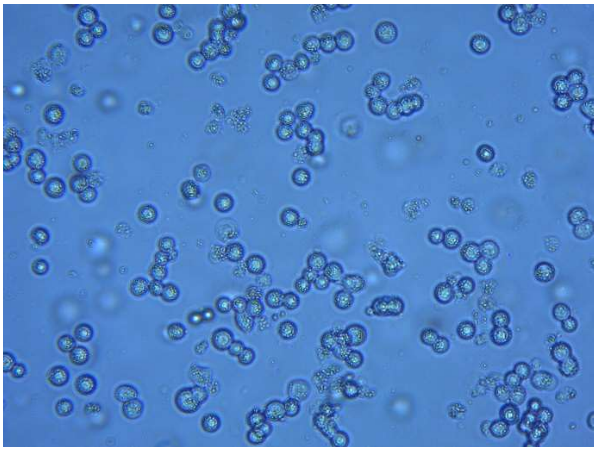

(B)

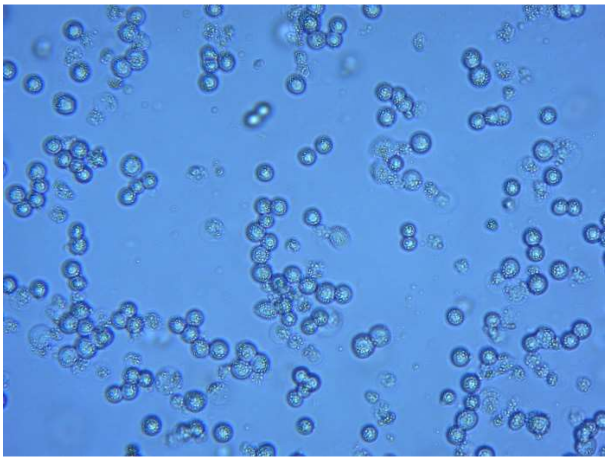

(C)

Figure S16. Photographs of A2780 cells upon their incubation during 24 h with solutions containing the RPMI 1640 media. (A) incubation with a solution not containing V^V anions; (B) upon incubation for 24 h with a ‘V_{10} solution? (C) upon incubation for 24 h with a ‘V_1 solution’. In (B) and (C) the [V]total was 50 μM, as used in the vanadium uptake experiments corresponding to Figure 9.

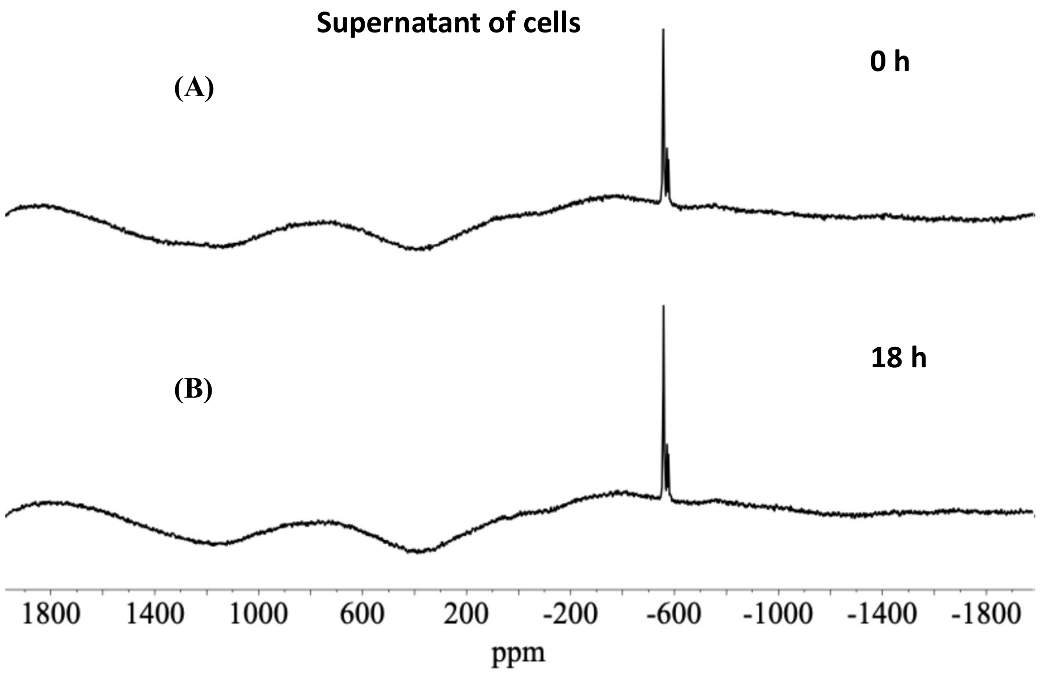

Figure S17. ^{51}V NMR spectra (A) of the supernatant of ‘V_{10} solution’, $[V]_{total}$ = 720 μM, added to RPMI medium with 2% FBS upon incubation for 3 h with A2780 cells. This spectrum was measured ~3 h after separation from cells (sample kept in ice) and it is here considered as t = 0 h. (B) ^{51}V NMR measured after 18 h keeping the same sample at room temperature. These spectra correspond to Figure 10 (B) and (C), respectively, but are drawn in a broader field range (ca. +2000 to -2000 ppm). The two spectra are basically equal and no peaks due to V_{10} anions are visible.

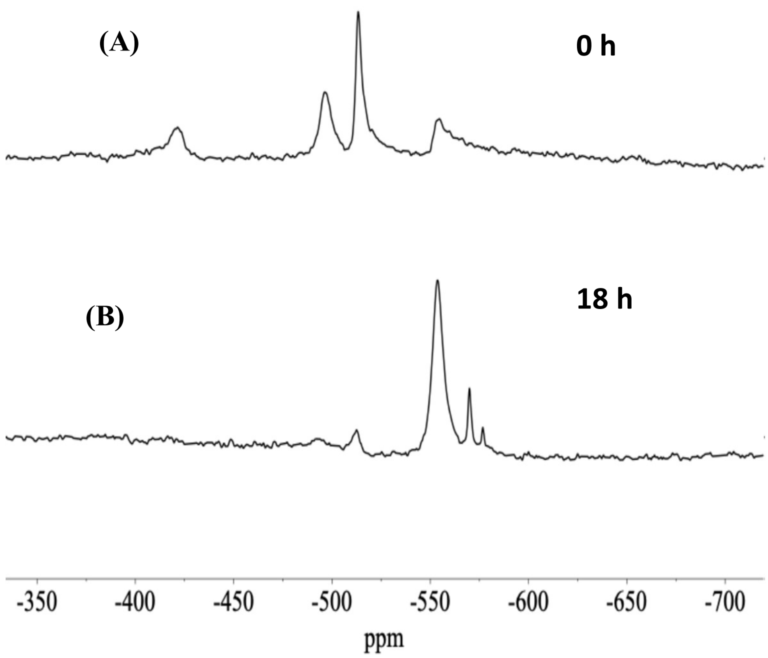

Figure S18. ^{51}V NMR spectra of a 'V_{10} solution' added to a RPMI incubation media of A2780 cells containing 2% FBS, so that $[V]_{total}$ = 800 μM. (A) Upon standing for ~3 h at 37 ºC and further placement of on ice at ~0ºC for about 3 h, the sample was removed from ice and the ^{51}V NMR spectrum was recorded at room temperature. (B) Spectrum recorded after keeping the same solution for additional ~18 h at room temperature. The peaks of decavanadates, visible at t = 0 ºC almost disappeared at t = 18 h (only the peak due to V10A at −512 ppm is clearly visible). The pH measured of this solution was 7.78.

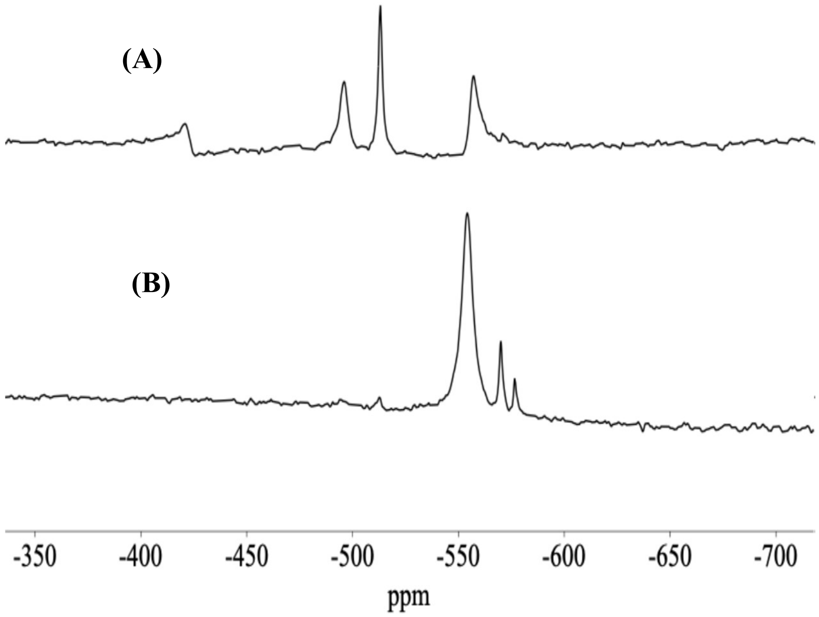

Figure S19. ^{51}V NMR spectrum of a ‘V_{10} solution’ with ($[V]_{total} \approx 800$ μM) added to a RPMI media (with no FBS). (A) Rather sharp and intense decavanadate peaks and V_1 peaks are visible; (B) after keeping the solution at RT for ~18 h the peaks due to V_{10} anions disappeared (a weak band at ~ −512 ppm is apparently visible, possibly due to V10A), and only sharp peaks due to V_1 (strong), V_2 and V_4 are seen.

References:

1. Macrae, C. F.; Sovago, I.; Cottrell, S. J.; Galek, P. T. A.; McCabe, P.; Pidcock, E.; Platings, M.; P. Shields, G.; Stevens, J. S.; Towler M.; Wood, P. A. Mercury 4.0: from visualization to analysis, design and prediction *J. Appl. Cryst*. **2020**, *53*, 226-235.

2. Brown, I. D.; Altermatt, D. Bond-valence parameters obtained from a systematic analysis of the Inorganic Crystal Structure Database. *Acta Crystallogr B* **1985**, *41*, 244-247.

3. O'Keefe, M.; Brese, N. E. Atom sizes and bond lengths in molecules and crystals, *J. Am. Chem. Soc.* **1991,** *113*, 3226-3229.

4. Palenik, R. C.; Abboud, K. A.; Palenik, G. J. Bond valence sums and structural studies of antimony complexes containing Sb bonded only to O ligands. *Inorg. Chim. Acta* **2005**, *358*, 1034-1040.
